# Nanomagnetic CoFe_2_O_4_@SiO_2_-EA-H_3_PO_4_ as a zwitterionic catalyst for the synthesis of bioactive pyrazolopyranopyrimidines and dihydropyrano[2,3-*c*]pyrazoles†

**DOI:** 10.1039/d3na00900a

**Published:** 2024-02-05

**Authors:** Ali Mirzaie, Lotfi Shiri, Mosstafa Kazemi, Nourkhoda Sadeghifard, Vahab Hassan Kaviar

**Affiliations:** a Department of Chemistry, Faculty of Basic Sciences, Ilam University P. O. Box 69315-516 Ilam Iran l.shiri@ilam.ac.ir; b Clinical Microbiology Research Center, Ilam University of Medical Sciences Ilam Iran

## Abstract

This study presents the development of a phosphoric acid-based zwitterionic catalyst immobilized on CoFe_2_O_4_ nanoparticles [CoFe_2_O_4_@SiO_2_-EA-H_3_PO_4_]. The structure of the nanocatalyst CoFe_2_O_4_@SiO_2_-EA-H_3_PO_4_ was identified by applying several spectroscopic techniques, *i.e.* FT-IR, SEM, TEM, XRD, EDX, elemental Mapping, VSM, TGA, and BET techniques. The catalytic efficiency of CoFe_2_O_4_@SiO_2_-EA-H_3_PO_4_ was evaluated in the water-based multicomponent synthesis of pyrazolopyranopyrimidine and dihydropyrano[2,3-*c*]pyrazole derivatives. Subsequently, an exploration of the antibacterial properties of the compounds was conducted. The catalytic system offers several advantages, encompassing high efficiency, brief reaction duration, uncomplicated operation, and facile recycling of the catalyst.

## Abstract 2

This study presents the development of a phosphoric acid-based zwitterionic catalyst immobilized on CoFe_2_O_4_ nanoparticles [CoFe_2_O_4_@SiO_2_-EA-H_3_PO_4_].

## Graphical abstract

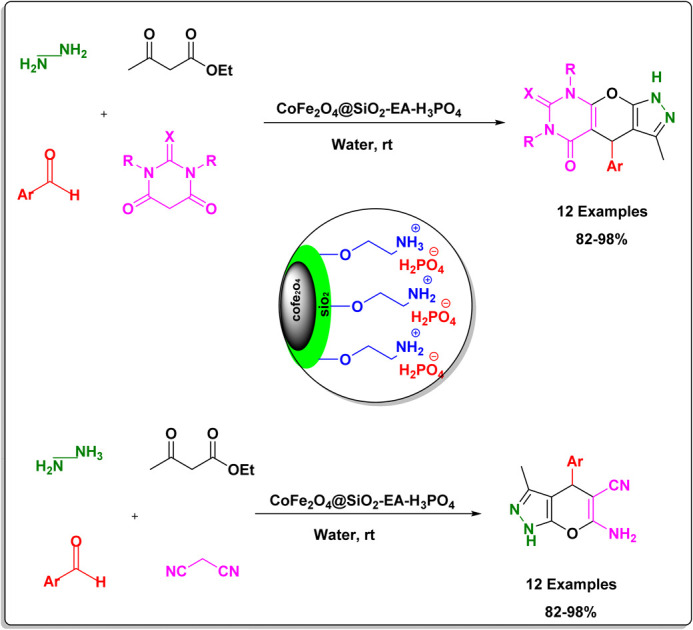

## 1. Introduction

Multicomponent reactions (MCRs) represent an exceedingly efficient approach for conducting diversity-oriented synthesis in the field of organic chemistry.^1–3^ In this sense, MCRs are recognized for their ability to create complex molecules in a single-pot reaction with high yields, short reaction times, minimal waste, and low purification costs. This makes MCRs a powerful tool used to construct novel and structurally diverse compounds.^4,5^

Compounds that include a nitrogen atom in their heterocyclic structure possess a variety of biological activities and pharmacological properties.^6–8^ Pyrazolopyranopyrimidine and dihydropyrano[2,3-*c*]pyrazoles exhibit notable promise in the realms of organic synthesis and medicine, owing to their prospective role as fundamental building blocks for pharmaceutical compounds.^9^ As a result, in order to synthesize such compounds, numerous techniques have been developed.^6,10^

Green catalysis, a subset of green chemistry, emphasizes the pressing need to develop and utilize catalysts that are environmentally friendly.^12,13^ In this regard, an ideal catalyst should be cost-effective, highly active, efficient, selective, stable, easily recoverable, and recyclable.^14^ Moreover, hybrid organic–inorganic materials have attracted considerable attention as heterogeneous catalysts in the field of organic synthesis.^15^ In recent years, there has been an increase in using heterogeneous catalysts due to their ability to be separated from the reaction mixture with ease, despite the potential lower activity as compared to homogeneous catalysts.^16,17^ Furthermore, the use of magnetic nanoparticles and their coated analogues has gained popularity due to their various advantages, *i.e.* effortless separation with the help of an external magnet, low toxicity, a highly active surface, easy recovery, and remarkable stability.^18^

Phosphoric acid stands out as one of the most crucial industrial catalysts, finding extensive application in a diverse range of organic transformations since 2004.^19^ Notably, various methods have been applied aiming to recycle phosphoric acid in these transformations.^20^ However, these methods are very costly and complex. It is worth mentioning that zwitterions are an efficient class of catalysts that include two different ions in their structure.^21^ As a part of our continuing efforts toward the development of solid acid catalysts, an efficient method is described for the preparation of a novel phosphoric acid-based zwitterionic catalyst immobilized on CoFe_2_O_4_ MNPs as the catalyst for the synthesis of important N-containing heterocycles in water and at room temperature.^22^ The overuse of antibiotics in the market has led to various health issues, including antibiotic resistance; therefore, discovering new drugs to combat microbial infections is crucial.^23^ The synthesized compounds were evaluated for their antibacterial properties against both Gram-negative and Gram-positive bacteria in our research.

## 2. Experimental

### 2.1. General methods

The chemicals and solvents utilized in this study were obtained from Merck and Sigma-Aldrich Chemical Companies and were used as received.

### 2.2. Typical method for the synthesis of the CoFe_2_O_4_@SiO_2_-EA-H_3_PO_4_ nanomagnetic catalyst

**Scheme 1:**
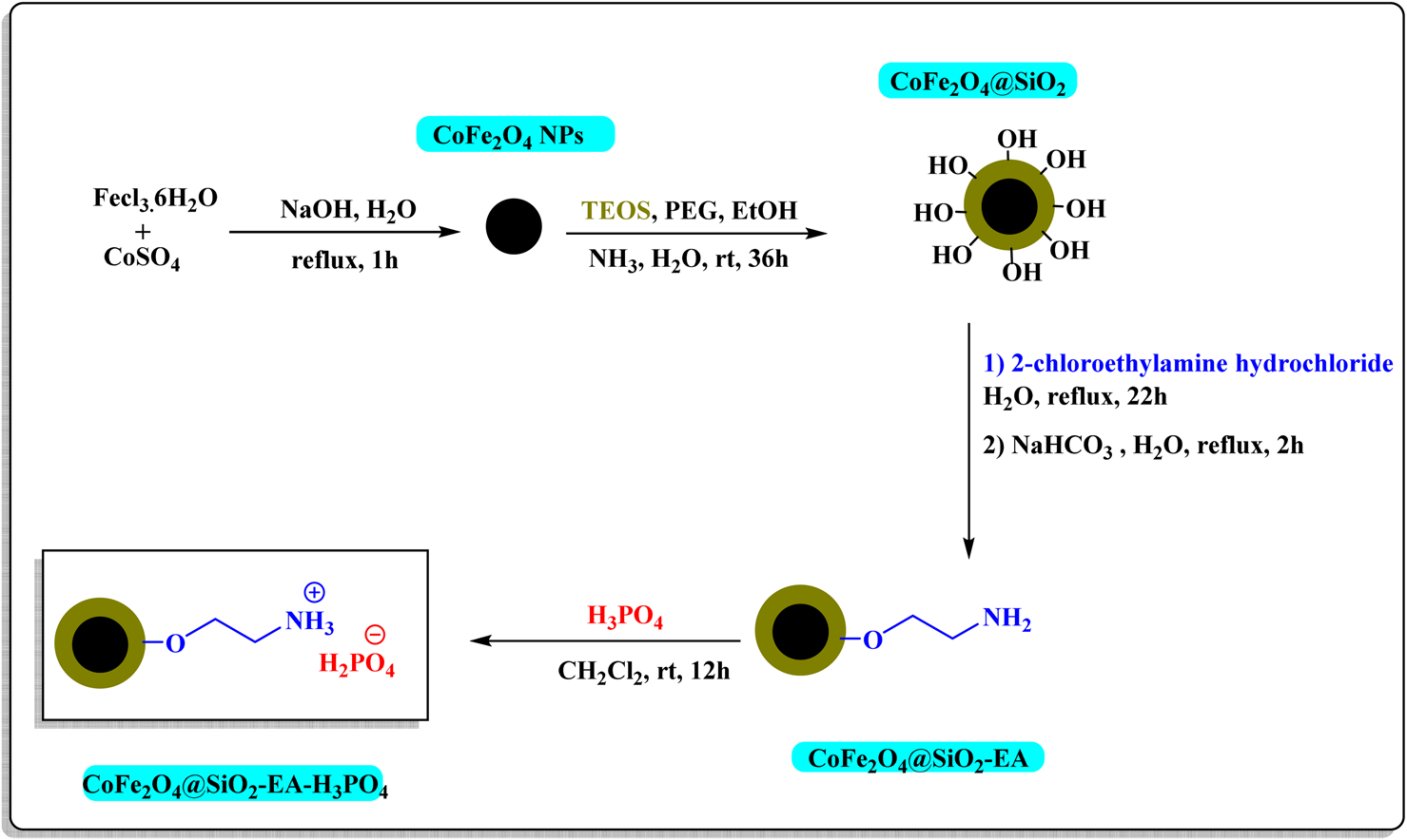
Stepwise synthesis of CoFe_2_O_4_@SiO_2_-EA-H_3_PO_4_.

The CoFe_2_O_4_@SiO_2_ core–shell was synthesized using the method that has been previously documented.^24^ In the subsequent step, 1.5 g of nanoparticles obtained from the previous step were dispersed in 50 mL of H_2_O for 10 min. The dispersion was then combined with 2 g of 2-chloroethylamine hydrochloride, followed by the addition of 1.5 g of NaHCO_3_. The mixture was subjected to reflux temperature for 24 h and 2 h, respectively. The resulting CoFe_2_O_4_@SiO_2_-EA product was extracted using a magnet, rinsed with DI water and, then, dried. In the concluding step, the CoFe_2_O_4_@SiO_2_-EA-MNP was dispersed in 10 mL of CH_2_Cl_2_ using ultrasound for 10 min. Subsequently, 1.5 mL of phosphoric acid was added dropwise to the reaction mixture, followed by stirring at room temperature. After 12 h, the nanoparticles were extracted with a magnet, washed with CH_2_Cl_2_ and EtOH, and then dried to produce the CoFe_2_O_4_@SiO_2_-EA-H_3_PO_4_ nanocatalyst outlined in Scheme 1.

### 2.3. The production of pyrazolopyranopyrimidines and dihydropyrano[2,3-*c*]pyrazole over the catalysis of CoFe_2_O_4_@SiO_2_-EA-H_3_PO_4_

A thoroughly stirred mixture comprising 1 mmol of ethyl acetoacetate and 1 mmol of hydrazine hydrate was supplemented with 1 mmol of barbituric acid or malononitrile, 1 mmol of aromatic aldehyde, and 20 mg of the catalyst. The reaction was monitored using TLC (applying n-hexane/ethyl acetate (1 : 2) as eluent) and, then, the mixture was diluted with hot ethanol. Subsequently, the catalyst was effortlessly removed using a magnet. Following that, the resulting substance which was subjected to recrystallization, using ethanol, was purified and, then, dried in an oven. The identification of the known compounds was done by comparing their melting points with those of authentic samples. Moreover, in certain instances, the analysis was conducted using FT-IR, ^1^H NMR, and ^13^C NMR, as illustrated in ESI Fig. S1–S33.†

### 2.4. Antibacterial activity

The cup plate technique was used to test the *in vitro* antimicrobial activity of the synthesized derivatives against Gram-negative *Escherichia coli* ATCC-25922 and Gram-positive *Staphylococcus aureus* ATCC-25923. The minimum inhibitory concentration (MIC) of the compounds was determined using the broth micro-dilution method, wherein concentrations of 1000 and 5000 μg were tested for all derivatives. The compounds exhibiting antibacterial activity were selected for MIC determination.

## 3. Results and discussion

Following the synthesis of CoFe_2_O_4_@SiO_2_-EA-H_3_PO_4_, the structure of the nanomagnetic catalyst was verified through the utilization of FT-IR, SEM, TEM, XRD, EDX, elemental Mapping, VSM, TGA, and BET techniques.

### 3.1. Characterization of CoFe_2_O_4_@SiO_2_-EA-H_3_PO_4_

#### 3.1.1. FT-IR studies

**Fig. 1:**
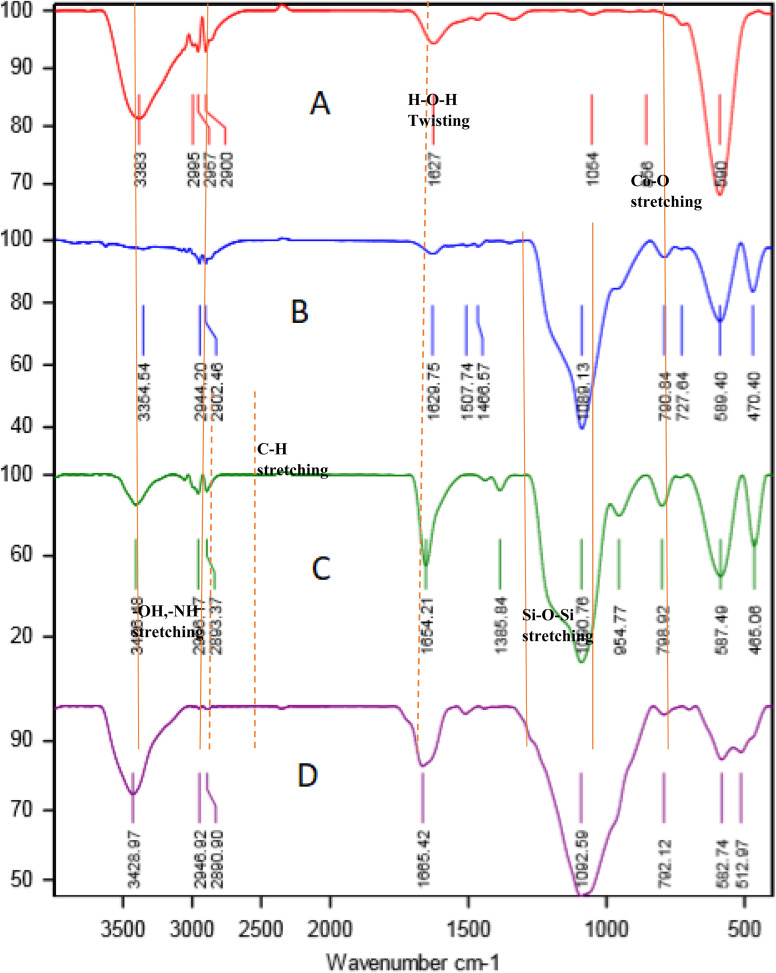
FT-IR spectra of CoFe_2_O_4_ (A), CoFe_2_O_4_@SiO_2_ (B), CoFe_2_O_4_@SiO_2_-EA (C), and CoFe_2_O_4_@SiO_2_-EA-H_3_PO_4_ (D).

The FT-IR spectra of CoFe_2_O_4_, CoFe_2_O_4_@SiO_2_, CoFe_2_O_4_@SiO_2_-EA, and CoFe_2_O_4_@SiO_2_-EA-H_3_PO_4_ MNPs are presented in Fig. 1. The Fe–O and O–H bands were identified through the observation of stretching vibrations at 590 cm^−1^ and 3383 cm^−1^, respectively, confirming the successful synthesis of CoFe_2_O_4_ MNP (Fig. 1A). The verification of the SiO_2_ content on the surface of CoFe_2_O_4_@SiO_2_ MNPs is confirmed by the stretching frequencies detected at 1089 cm^−1^ (Fig. 1B). The grafting of 2-chloroethylamine on the CoFe_2_O_4_@SiO_2_ surface is verified by distinct peaks for methylene groups (CH_2_) at 2897–2949 cm^−1^ and a broadening peak at 3435 cm^−1^ for the NH_2_ group (Fig. 1C). Furthermore, the broad peak at around 3000–3700 cm^−1^ corresponds to the ammonium content of the catalyst, while symmetric and asymmetric PO stretching modes are observed at 1200 cm^−1^ (Fig. 1D).

#### 3.1.2. Scanning electron microscopy (SEM) studies

**Fig. 2:**
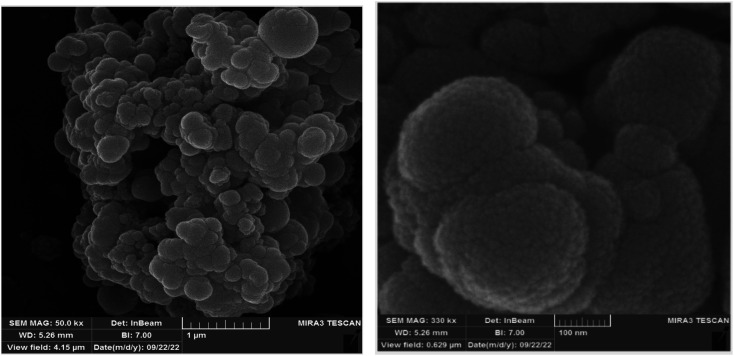
SEM image of the CoFe_2_O_4_@SiO_2_-EA-H_3_PO_4_ nanocatalyst.

The SEM image of CoFe_2_O_4_@SiO_2_-EA-H_3_PO_4_ MNPs is shown in Fig. 2. The obtained images indicate that the nanoparticles are spherical and fall within the size range of nanomaterials.

#### 3.1.3. Transmission electron microscopy (TEM) studies

**Fig. 3:**
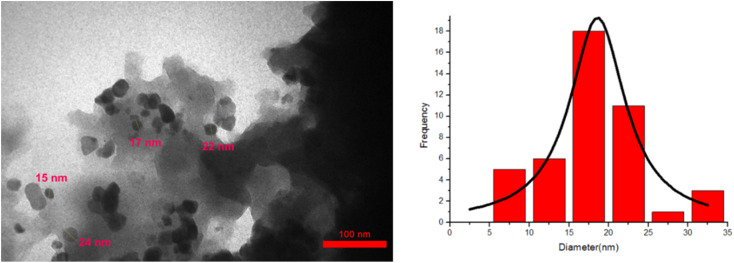
TEM micrograph of the CoFe_2_O_4_@SiO_2_-EA-H_3_PO_4_ nanocatalyst.

Transmission electron microscopy (TEM) was used to examine the morphology, size and shape of the CoFe_2_O_4_@SiO_2_-EA-H_3_PO_4_ MNPs. The TEM images indicate that the catalyst has a spherical form and a core–shell structure, with the average particle size at around 15–20 nm which is illustrated in Fig. 3. Furthermore, the TEM results were consistent with those obtained from SEM images.

#### 3.1.4. XRD analysis

**Fig. 4:**
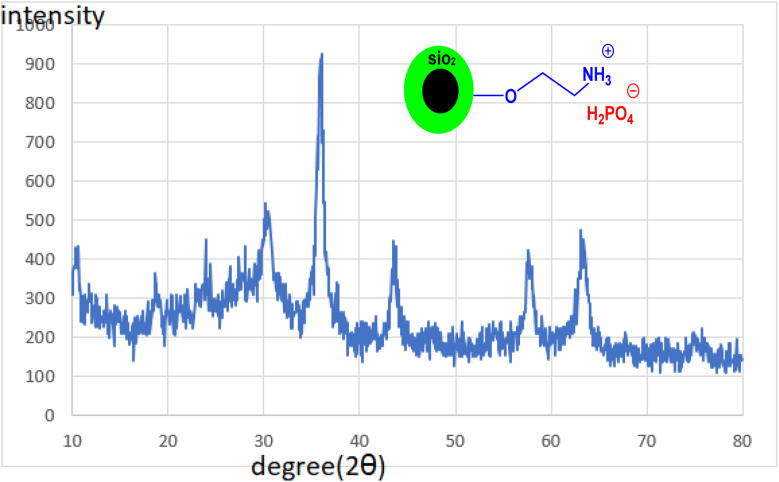
XRD patterns of CoFe_2_O_4_@SiO_2_-EA-H_3_PO_4_ MNPs.

Fig. 4 depicts the XRD pattern of CoFe_2_O_4_@SiO_2_-EA-H_3_PO_4_ MNPs. The plots exhibit six peaks at 2*θ* = 30.21°, 35.76°, 43.36°, 54.06°, 57.61° and 63.16°, which correspond to the standard plot of CoFe_2_O_4_ MNPs.^25^ The findings validate that the CoFe_2_O_4_ crystal structure remains unaltered even after the addition of organic and inorganic layers onto its surface. Through the application of Debye–Scherrer's calculation, it has been established that the nanoparticles exhibit a dimension of approximately 17.43 nm, aligning closely with the findings from TEM analysis.

#### 3.1.5. EDX analysis

**Fig. 5:**
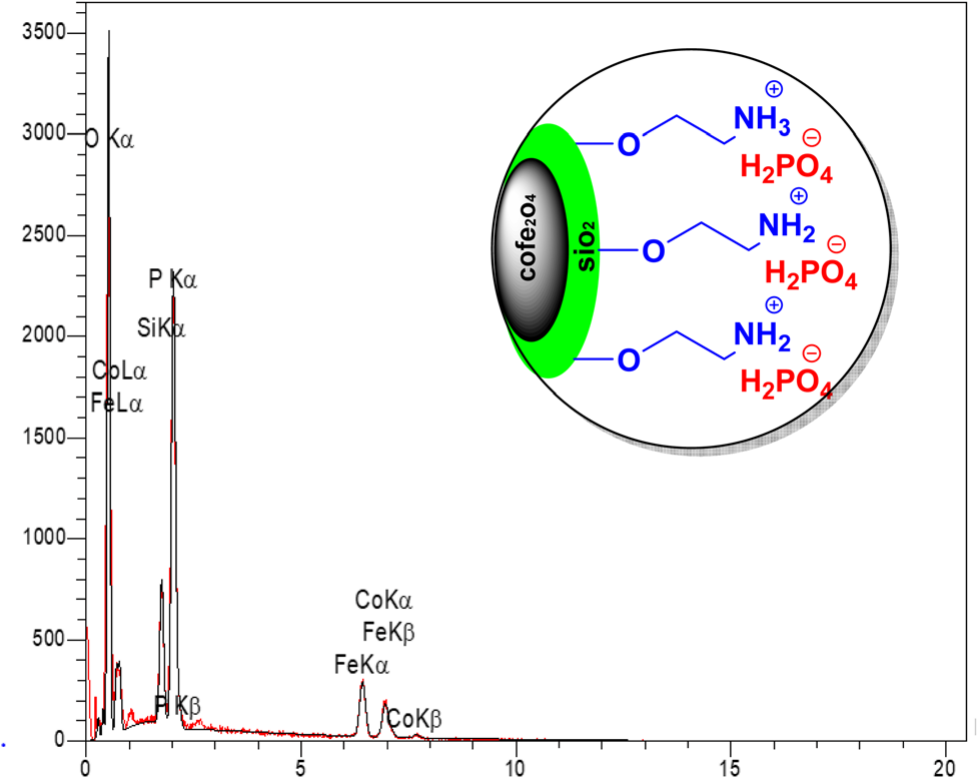
EDX spectrum of CoFe_2_O_4_@SiO_2_-EA-H_3_PO_4_ MNPs.

To determine the elemental composition of the CoFe_2_O_4_@SiO_2_-EA-H_3_PO_4_ catalyst, EDX spectroscopy was employed. The analysis confirmed the presence of Fe, Co, O, Si, C, P, and N elements, as predicted (Fig. 5).

#### 3.1.6. EDX elemental mapping analysis

**Fig. 6:**
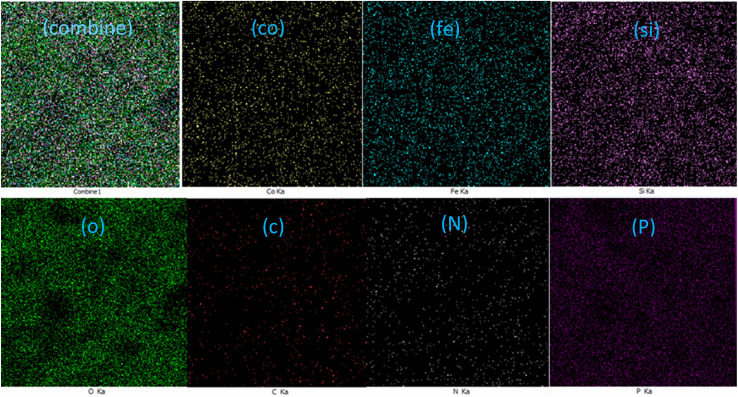
Displaying the scattering of elements in CoFe_2_O_4_@SiO_2_-EA-H_3_PO_4_ MNPs.

EDX mapping imaging was used to examine the elemental composition of the CoFe_2_O_4_@SiO_2_-EA-H_3_PO_4_ MNP catalyst. The analysis revealed a significant amount of cobalt, iron, silicon, and oxygen elements in the CoFe_2_O_4_@SiO_2_ material. Significantly, the distribution of carbon, nitrogen, and phosphorus confirmed the presence of the desired zwitterion functionalities (EA-H_3_PO_4_) on the nanomagnetic support surface, as illustrated in Fig. 6.

#### 3.1.7. VSM analysis

**Fig. 7:**
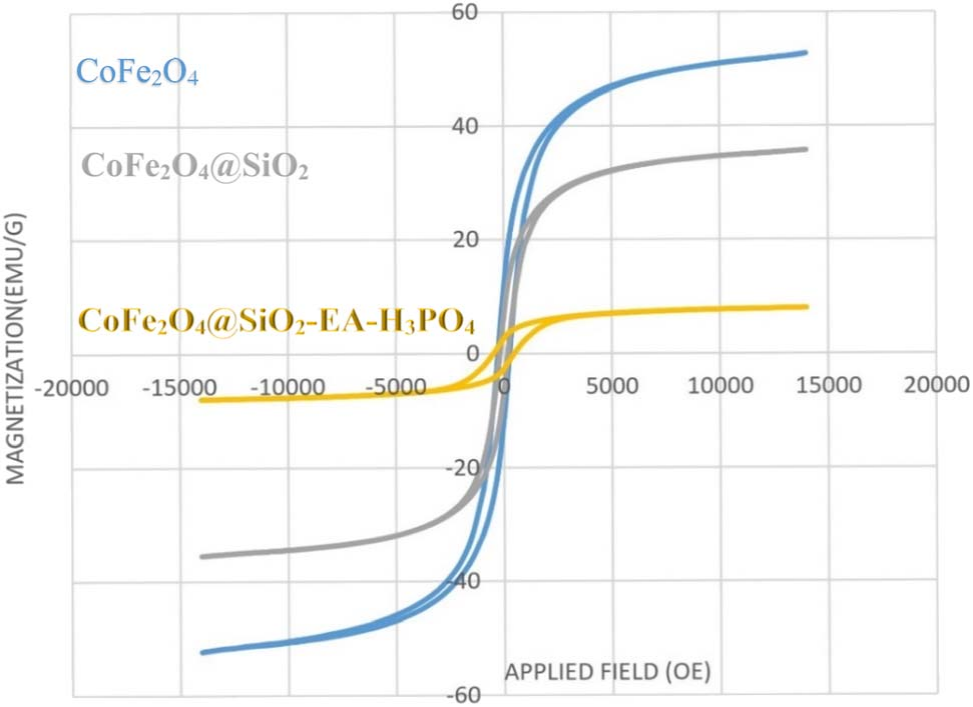
VSM analysis of CoFe_2_O_4_ MNPs (blue curve), CoFe_2_O_4_@SiO_2_ (gray curve) and CoFe_2_O_4_@SiO_2_-EA-H_3_PO_4_ (yellow curve) nanocatalysts.

The VSM technique was utilized to examine the magnetic characteristics of CoFe_2_O_4_, CoFe_2_O_4_@SiO_2_, and CoFe_2_O_4_@SiO_2_-EA-H_3_PO_4_ MNPs (as shown in Fig. 7). The magnetic potency of each material was assessed and determined to be 53 emu g^−1^, 35 emu g^−1^ and 9 emu g^−1^, respectively. This decrease in saturation magnetization indicates successful incorporation of the amorphous silica phase and organic functionalities onto the CoFe_2_O_4_ surface.

#### 3.1.8. TGA analysis

**Fig. 8:**
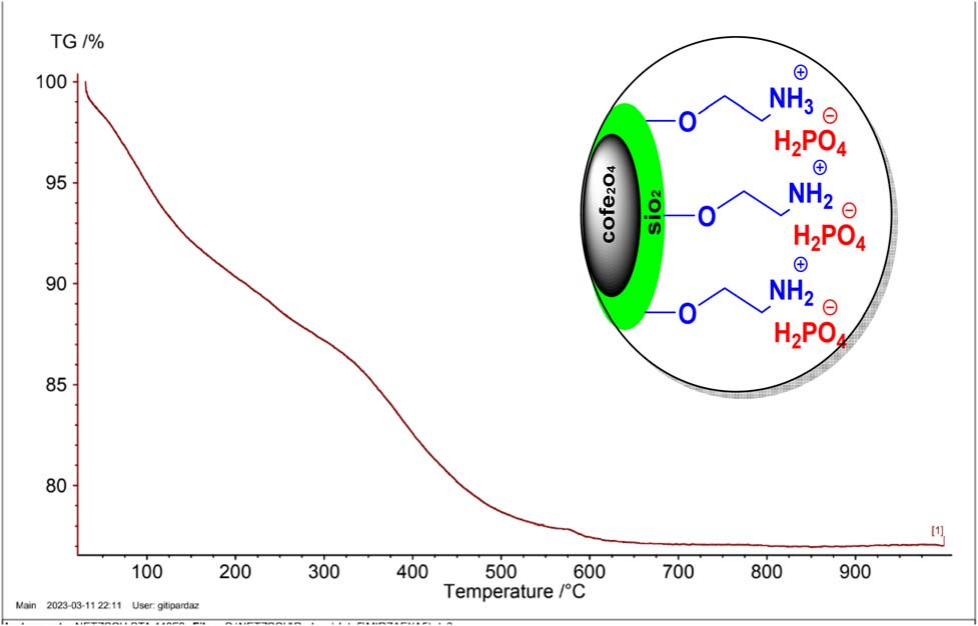
TGA curve of CoFe_2_O_4_@SiO_2_-EA-H_3_PO_4_ MNPs.

Fig. 8 displays the TGA curve for the CoFe_2_O_4_@SiO_2_-EA-H_3_PO_4_ catalyst, revealing three distinct phases of weight loss. The initial mass reduction at room temperature up to approximately 200 °C is attributed to the elimination of residual solvents from the catalyst preparation process. Furthermore, the significant mass decrease observed between 200 and 590 °C, encompassing the second and third stages of weight loss, is likely attributed to the decomposition of both organic and inorganic components associated with the CoFe_2_O_4_ nanoparticles.

#### 3.1.9. N_2_ adsorption/desorption analysis

**Fig. 9:**
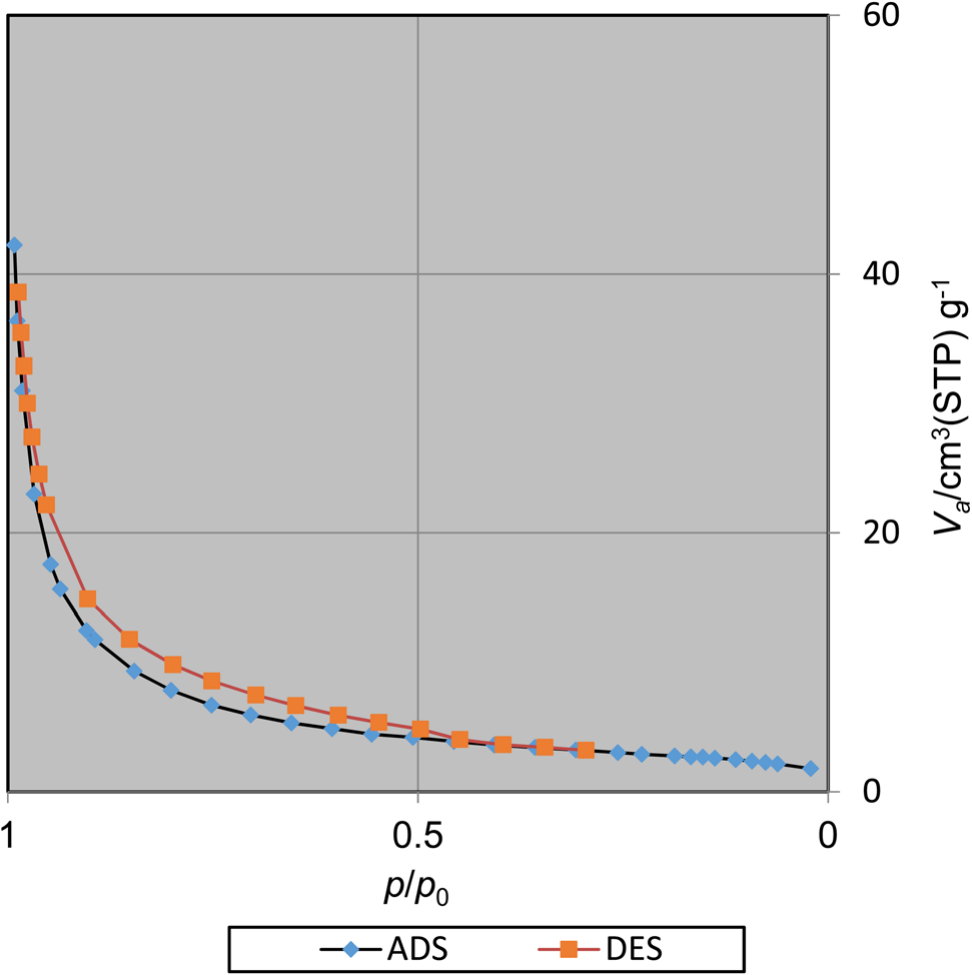
N_2_ adsorption/desorption isotherms of CoFe_2_O_4_@SiO_2_-EA-H_3_PO_4_ MNPs.

The surface area and pore volume of CoFe_2_O_4_@SiO_2_-EA-H_3_PO_4_ were studied applying the Brunauer–Emmett–Teller (BET) method (Fig. 9). The BET specific surface area (SSA) and total pore volume (TPV) were ascertained to be 110.31 m^2^ g^−1^ and 2.36 cm^3^ g^−1^, respectively, which indicate a high surface area for catalysis applications.

### 3.2. Catalysis study

Our research endeavors center around the development of efficient synthetic pathways for heterocycles, employing magnetic nanoparticle catalysts. Using CoFe_2_O_4_@SiO_2_-EA-H_3_PO_4_ MNPs as the catalyst, a one-pot, four-component reaction was conducted that involved ethylacetoacetate, hydrazine hydrate, barbituric acid, and aromatic aldehydes to produce pyrazolopyranopyrimidines. The reaction conditions were optimized through studying the model reaction while using 4-chlorobenzaldehyde and varying the solvent type and amount of the catalyst. The best reaction conditions were achieved using 20 mg of the catalyst in H_2_O solvent at room temperature (entry 3, Table 1).

**Table 1:** Effect of different amounts of catalyst and solvent on the model reaction^a^

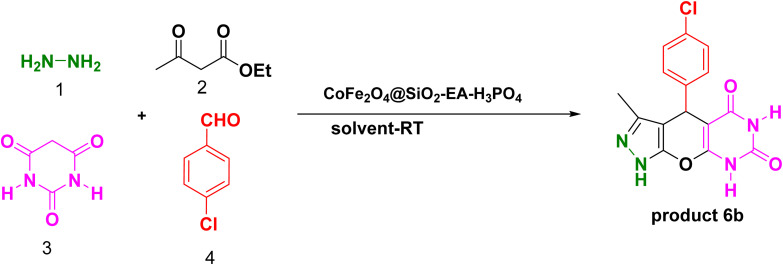					
Entry	Catalyst	Amount of catalyst (mg)	Solvent (mL)	Time (min)	Yield^b^ (%)
1	CoFe_2_O_4_@SiO_2_-EA-H_3_PO_4_	10	H_2_O	5	80
2	CoFe_2_O_4_@SiO_2_-EA-H_3_PO_4_	15	H_2_O	5	85
**3**	**CoFe**_**2**_ **O**_**4**_ **@SiO**_**2**_ **-EA-H**_**3**_ **PO**_**4**_	**20**	**H**_**2**_ **O**	**5**	**98**
4	CoFe_2_O_4_@SiO_2_-EA-H_3_PO_4_	30	H_2_O	5	98
5	CoFe_2_O_4_@SiO_2_-EA-H_3_PO_4_	20	EtOH	15	80
6	CoFe_2_O_4_@SiO_2_-EA-H_3_PO_4_	20	H_2_O/EtOH (1 : 1)	15	85
7	CoFe_2_O_4_@SiO_2_-EA-H_3_PO_4_	20	DMF	15	80
8	CoFe_2_O_4_@SiO_2_-EA-H_3_PO_4_	20	CH_3_CN	15	75
9	CoFe_2_O_4_@SiO_2_-EA-H_3_PO_4_	20	EtOAC	20	50
10	CoFe_2_O_4_@SiO_2_-EA	20	H_2_O	20	76
11	CoFe_2_O_4_@SiO_2_	20	H_2_O	20	60
12	CoFe_2_O_4_	20	H_2_O	20	50
13	—	—	H_2_O	60	10

a Reaction conditions: ethyl acetoacetate (1.0 mmol), hydrazine hydrate (1.0 mmol), 4-chlorobenzaldehyde (1.0 mmol) and barbituric acid (1.0 mmol) catalyst (g) and solvent (2 mL).

b Optimum conditions.

Table 2 presents the results of synthesizing pyrazolopyranopyrimidine derivatives using different benzaldehyde and barbituric acid combinations under optimized reaction conditions, resulting in good yields and short reaction times. The synthesis of these derivatives was influenced by both electron-withdrawing and electron-releasing groups. The synthesized derivatives using benzaldehydes, featuring electron-withdrawing groups, exhibited shorter synthesis times and higher yields in comparison to those with electron-donating groups.

**Table 2:** Synthesis of pyrazolopyranopyrimidine derivatives catalyzed by CoFe_2_O_4_@SiO_2_-EA-H_3_PO_4_^a^

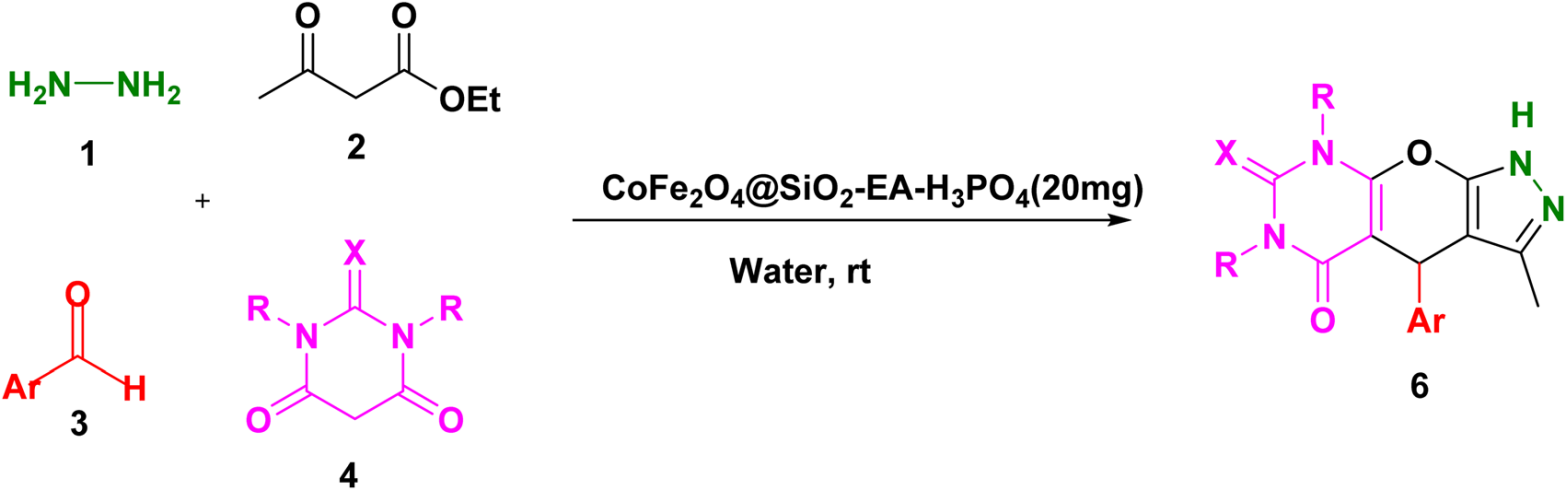							
Entry	Ar	R	X	Product	Time (min)	Yield^b^ (%)	MP (°C)
1	C_6_H_5_	H	O	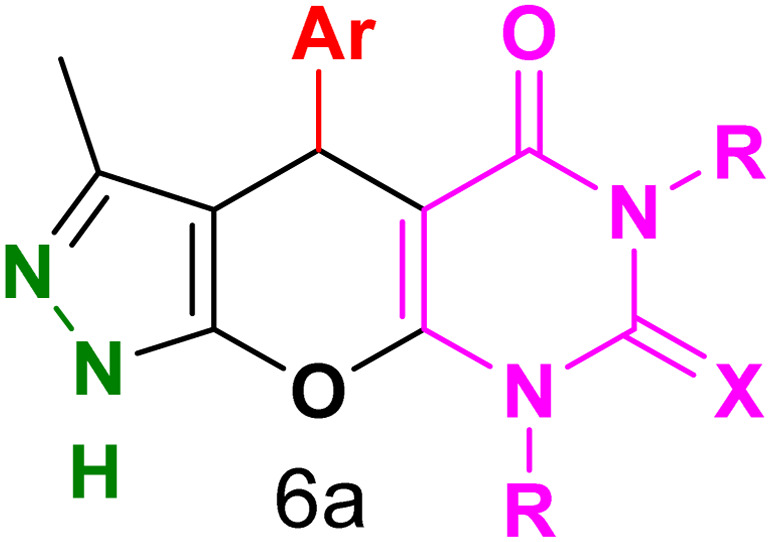	10	98	214–216 (ref. 10)
2	4-ClC_6_H_4_	H	O	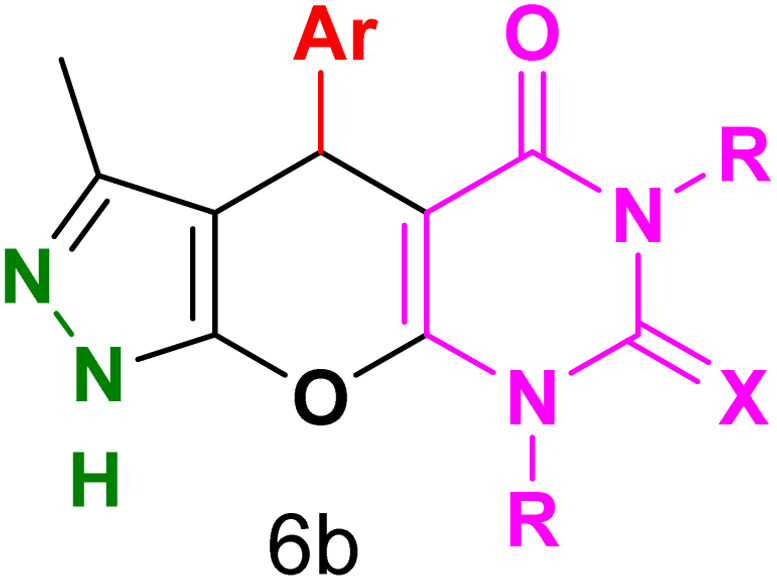	5	98	216–218 (ref. 10)
3	2-ClC_6_H_4_	H	O	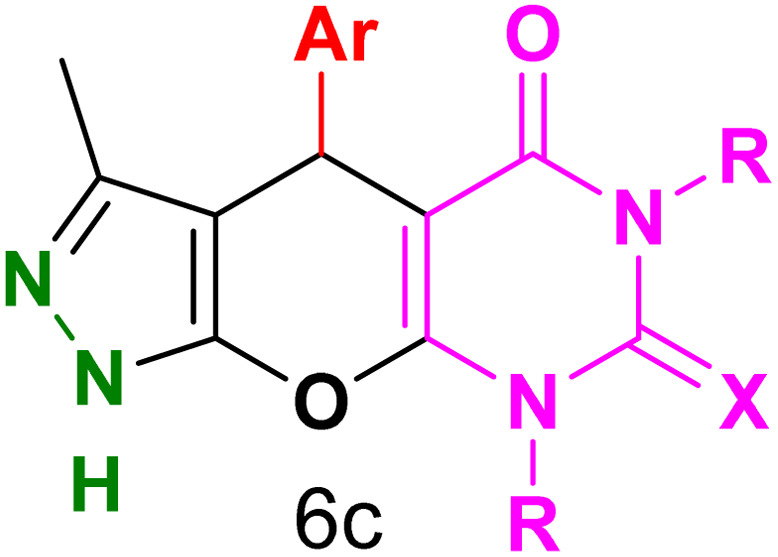	10	96	228–230 (ref. 10)
4	4-MeC_6_H_4_	H	O	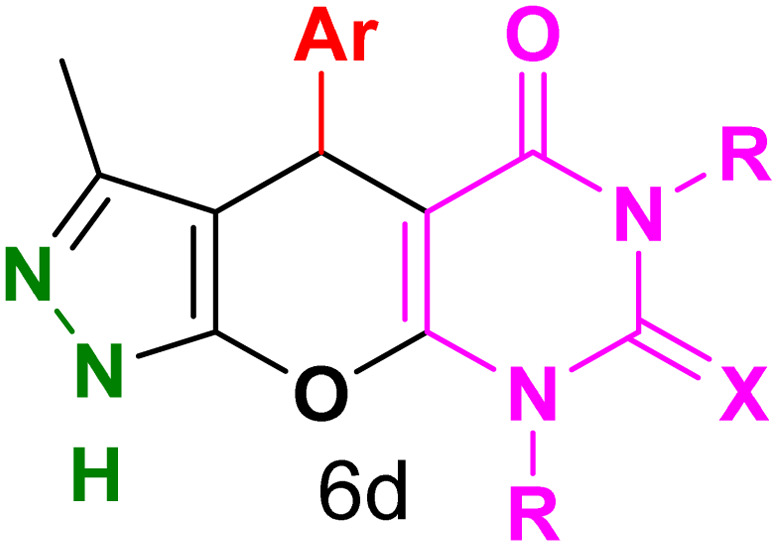	15	85	200–202 (ref. 10)
5	2-OMeC_6_H_4_	H	O	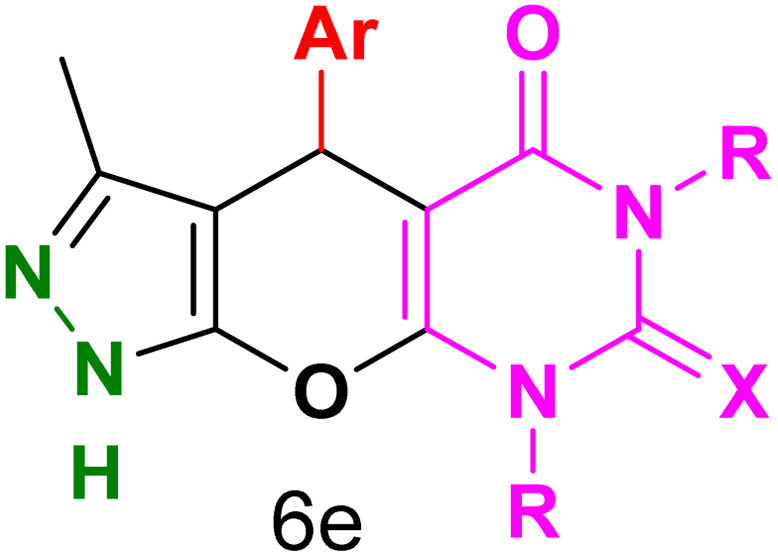	20	91	230–231 (ref. 10)
6	4-OMeC_6_H_4_	H	O	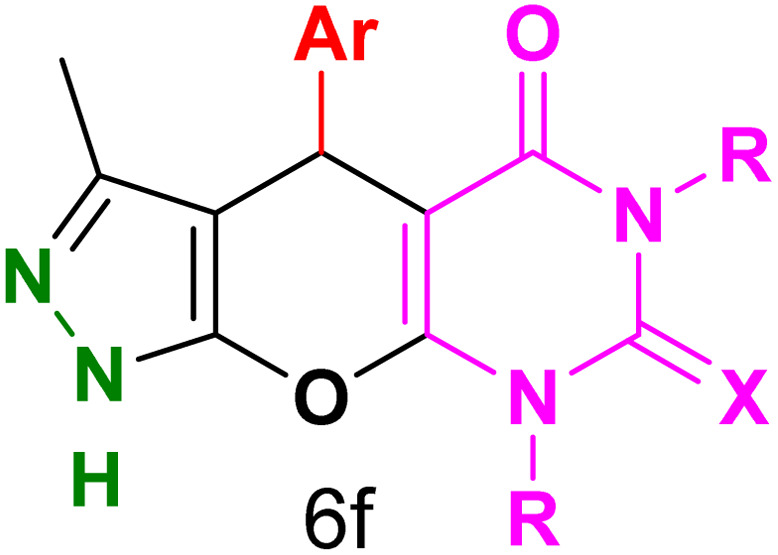	15	97	225–227 (ref. 10)
7	2-ClC_6_H_4_	H	S	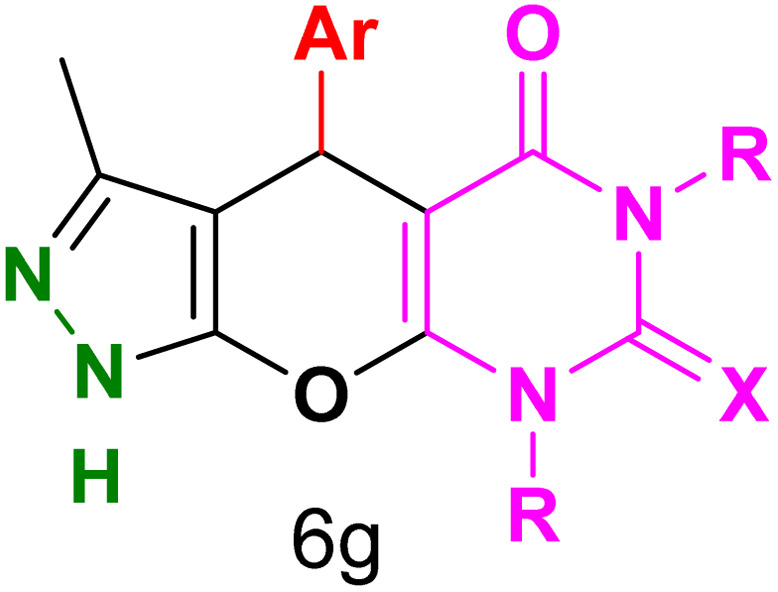	10	85	170–172 (ref. 10)
8	4-NO_2_C_6_H_4_	H	S	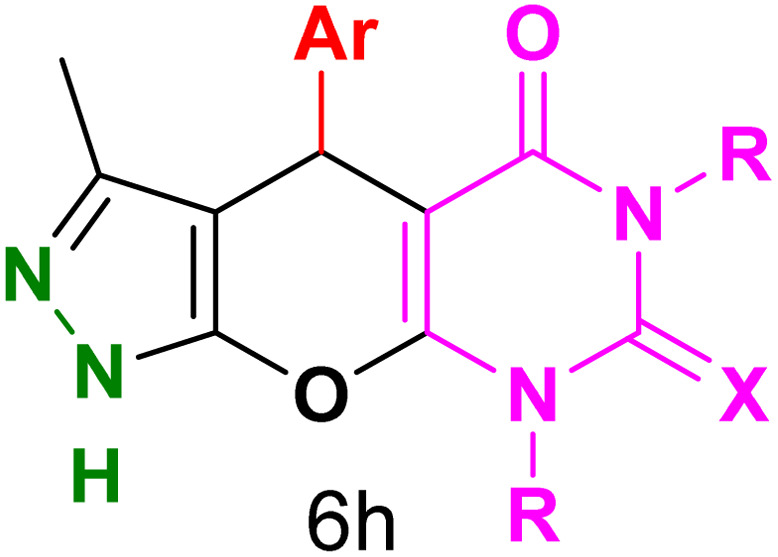	5	85	230–232 (ref. 26)
9	4-OMeC_6_H_4_	H	S	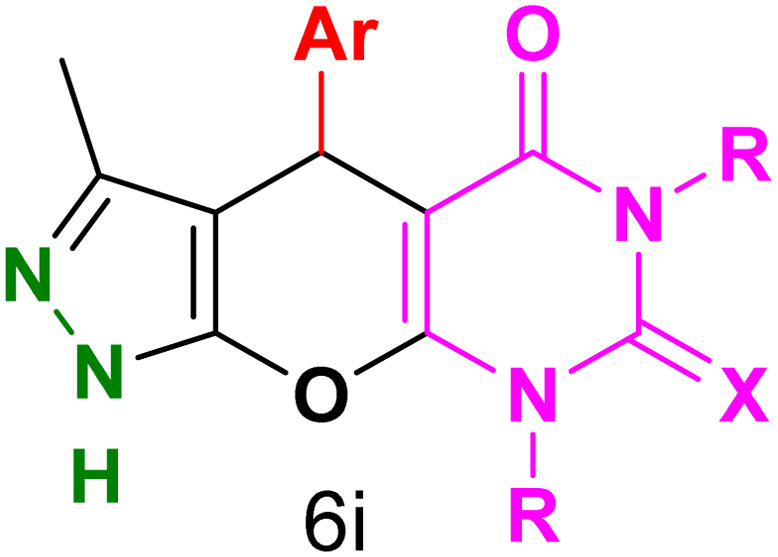	15	86	226–228 (ref. 10)
10	C_6_H_5_	CH_3_	O	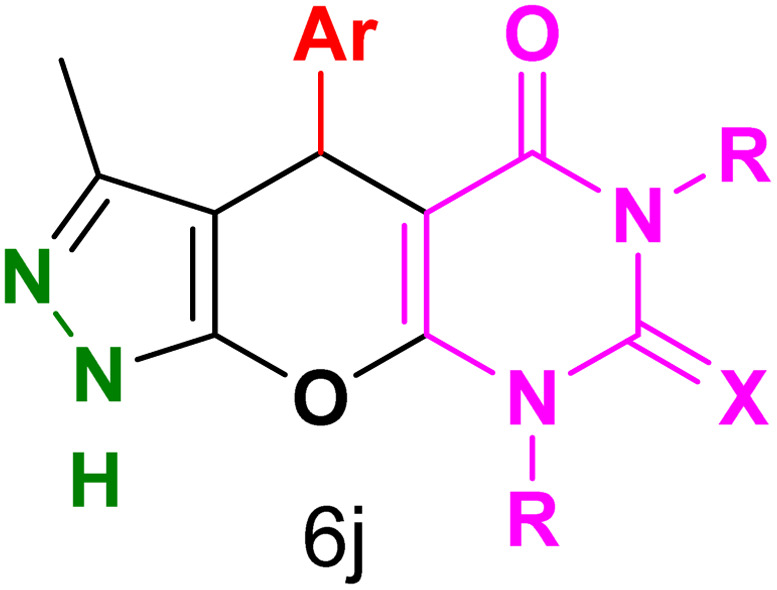	25	86	192–194 (ref. 10)
11	4-ClC_6_H_4_	CH_3_	O	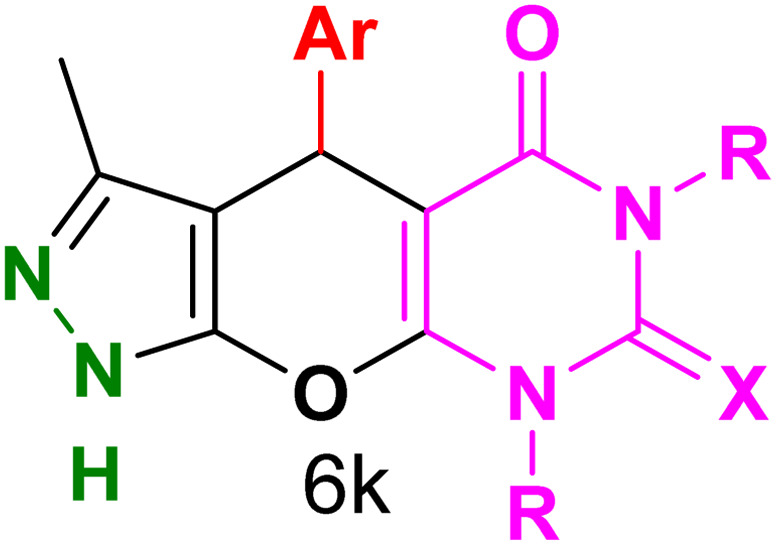	20	87	200–202 (ref. 10)
12	4-MeC_6_H_4_	CH_3_	O	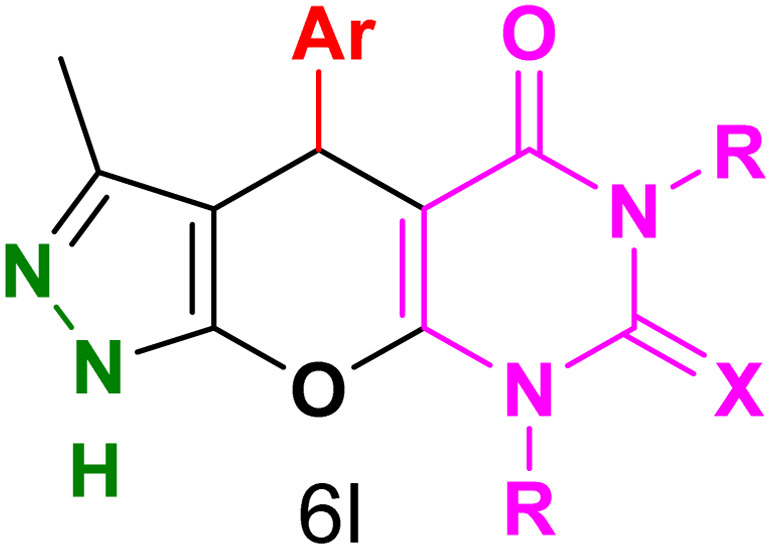	25	82	173–175 (ref. 10)

a Reaction conditions: ethyl acetoacetate (1.0 mmol), hydrazine hydrate (1.0 mmol), benzaldehyde (1.0 mmol), barbituric acid (1.0 mmol) and catalyst (20 mg) in 2 mL H_2_O.

b Isolated yield.

To explore the scope of the reaction, malononitrile was used in place of barbituric acid to synthesize dihydropyrano[2,3-*c*]pyrazole products. The optimal reaction conditions, previously established for pyrazolopyranopyrimidines, were applied to synthesize various dihydropyrano[2,3-*c*]pyrazole compounds, as detailed in Table 3. The findings indicate that the catalyst employed in this study consistently produces dihydropyrano[2,3-*c*]pyrazole products with exceptional yields and rapid reaction times.

**Table 3:** Synthesis of dihydropyrano[2,3-*c*]pyrazoles catalyzed by CoFe_2_O_4_@SiO_2_-EA-H_3_PO_4_^a^

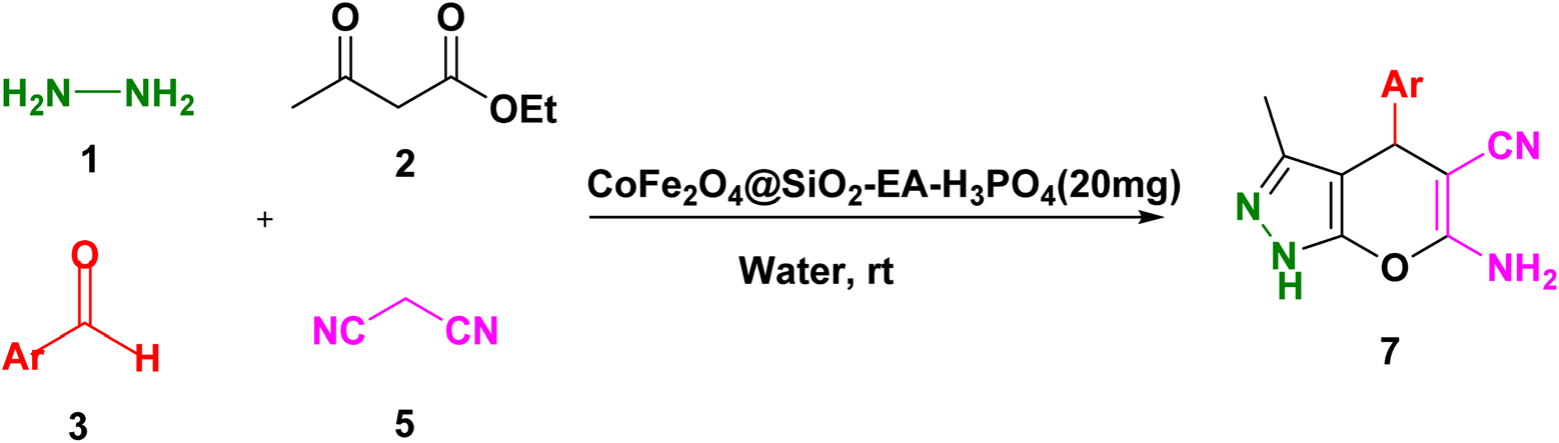					
Entry	Ar	Product	Time (min)	Yield^b^ (%)	MP (°C)
1	C_6_H_5_	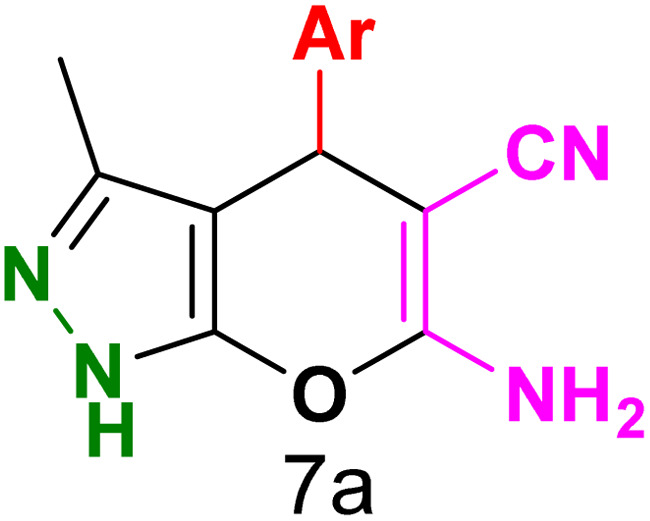	10	98	244–246 (ref. 11)
2	4-ClC_6_H_4_	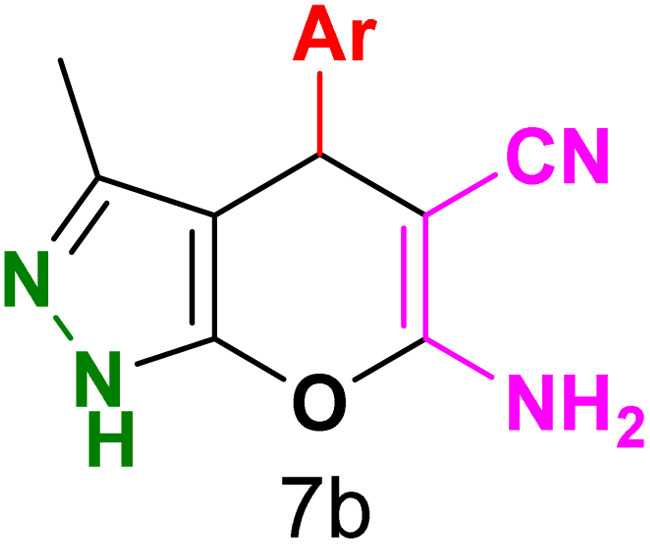	5	98	232–234 (ref. 11)
3	2-ClC_6_H_4_	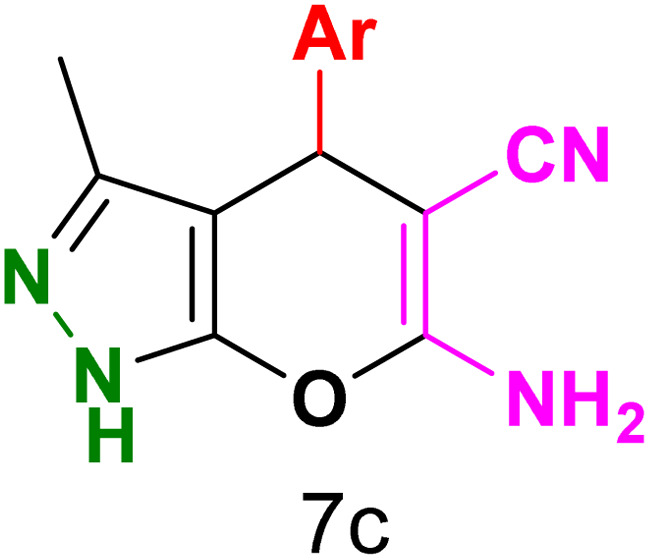	10	96	245–247 (ref. 27)
4	4-MeC_6_H_4_	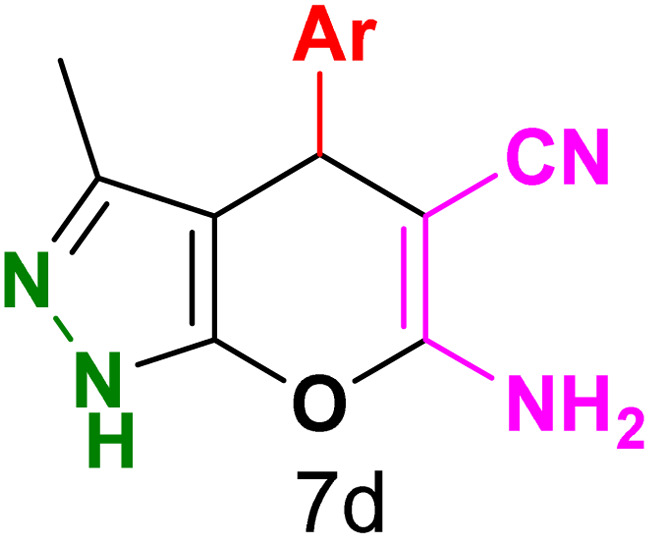	15	85	195–197 (ref. 28)
5	2-OMeC_6_H_4_	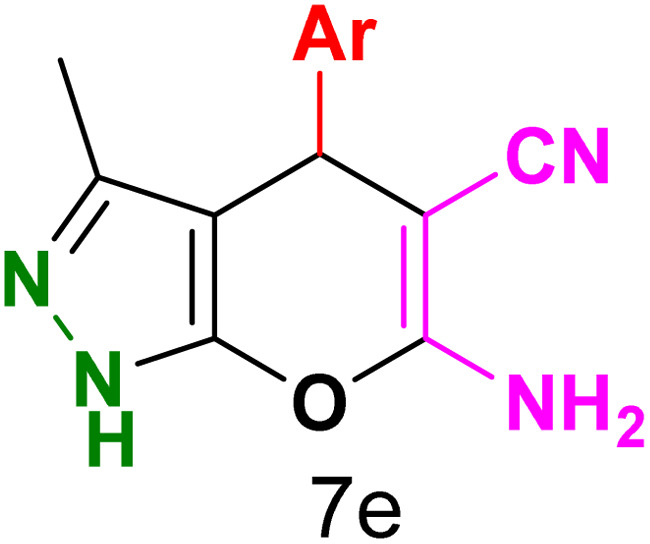	25	91	221–223 (ref. 29)
6	4-OMeC_6_H_4_	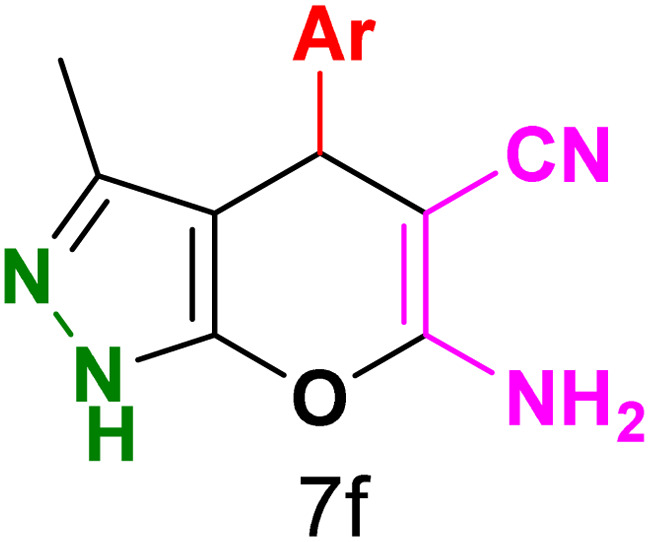	20	97	211–213 (ref. 28)
7	4-OHC_6_H_4_	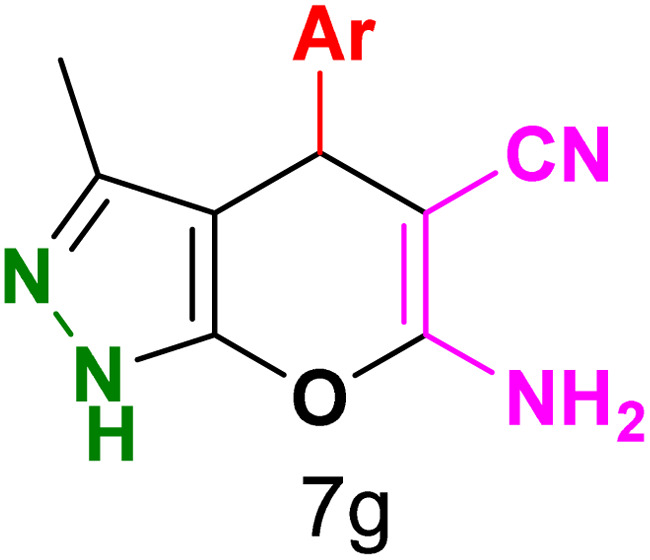	25	85	224–226 (ref. 11)
8	3-OHC_6_H_4_	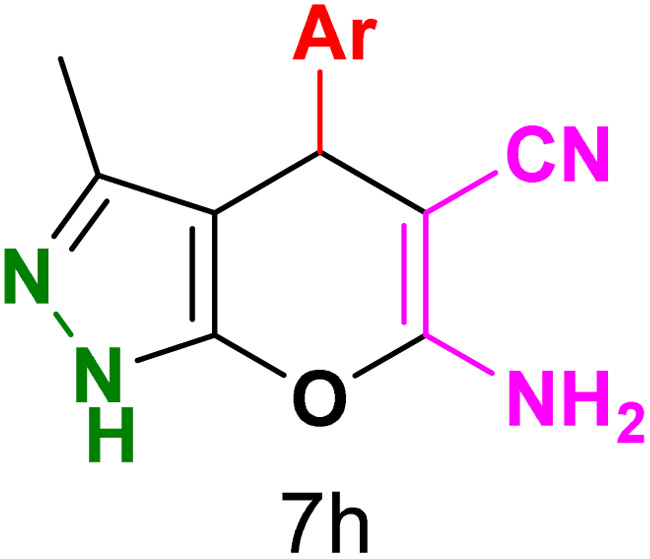	15	85	246–248 (ref. 30)
9	4-N(Me)_2_C_6_H_4_	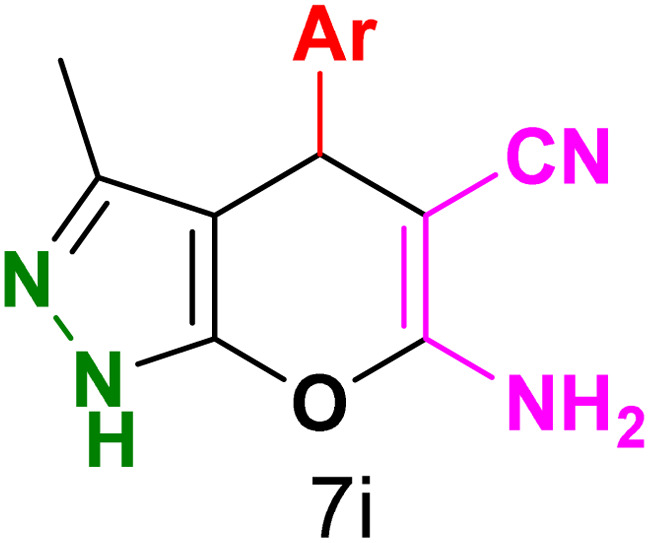	30	86	170–172 (ref. 28)
10	3-NO_2_ C_6_H_4_	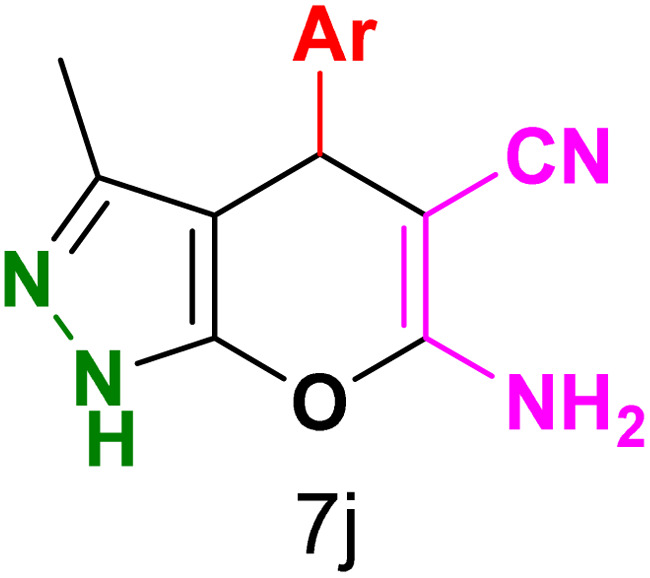	15	86	233–235 (ref. 11)
11	4-NO_2_ C_6_H_4_	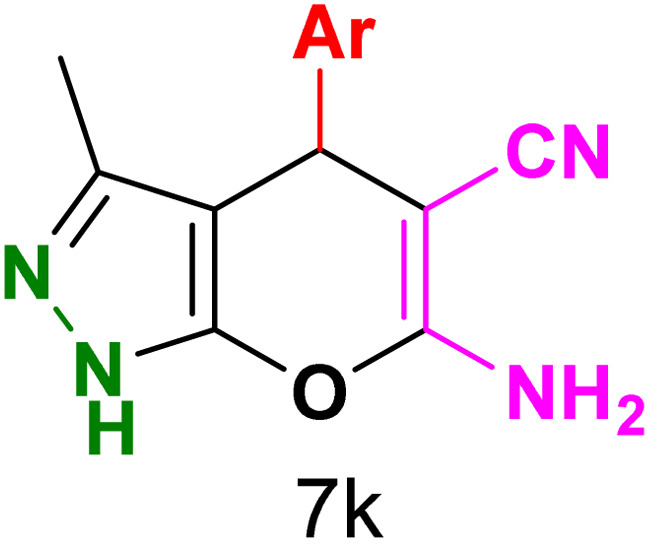	5	87	249–251 (ref. 11)
12	4-Br C_6_H_4_	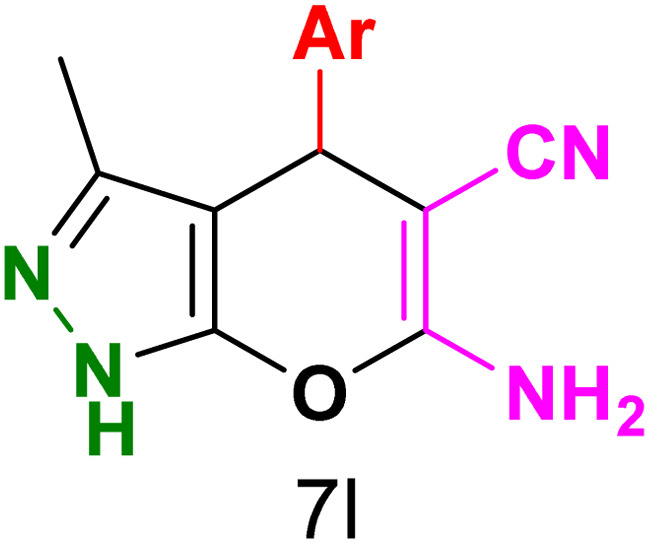	10	82	247–249 (ref. 11)

a Reaction conditions: ethyl acetoacetate (1.0 mmol), hydrazine hydrate (1.0 mmol), benzaldehyde (1.0 mmol), malononitrile (1.0 mmol) and catalyst (20 mg) in 2 mL water.

b Isolated yield.

### 3.3. Plausible mechanism for the synthesis of pyrazolopyranopyrimidine and dihydropyrano[2,3-*c*]pyrazoles

**Scheme 2:**
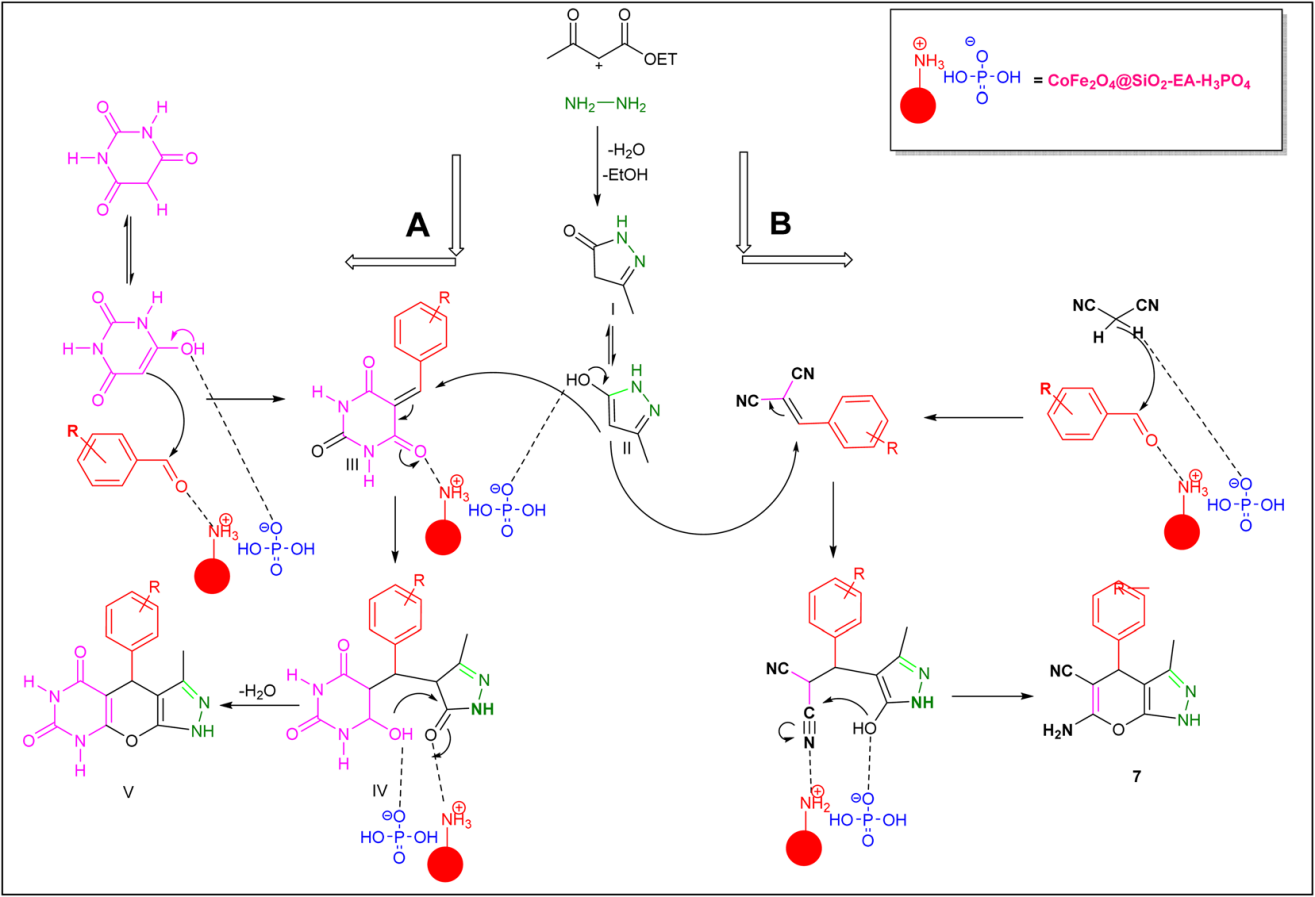
A possible mechanism for the synthesis of pyrazolopyranopyrimidine and dihydropyrano[2,3-*c*]pyrazoles catalyzed by the CoFe_2_O_4_@SiO_2_-EA-H_3_PO_4_ nanocomposite.

A plausible mechanism for the synthesis of pyrazolopyranopyrimidine, based on the previously reported reactions, is shown in Scheme 2. Initially, the condensation of ethyl acetoacetate with hydrazine resulted in the formation of pyrazole (I), which can subsequently be converted to its corresponding enolate form (II). In the presence of the nanocatalyst, Knoevenagel condensation takes place between benzaldehyde and barbituric acid, leading to the formation of intermediate (III). Subsequently, compound (II) reacts with intermediate (III) through a Michael addition, resulting in the formation of intermediate (IV). Ultimately, through the reaction of the intramolecular intermediate (IV) and the elimination of a water molecule, compound 5a was synthesized. The mechanism for the formation of dihydropyrano[2,3-*c*]pyrazoles closely parallels the synthesis mechanism of pyrazolopyranopyrimidine, with the substitution of malononitrile for barbituric acid.^30^

### 3.4. Antibacterial activity

**Fig. 10:**
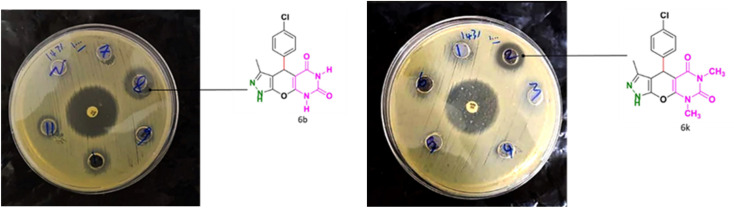
The antibacterial activity of synthesized derivatives against Gram positive bacteria (*S. aureus*).

Table 4 and Fig. 8 show the antibacterial results of pyrazolopyranopyrimidine derivatives, with compounds 6b, 6c, 6e, 6f and 6k being the most active antibacterial agents. The structure–activity relationship revealed that amide groups at the pyrimidine ring contribute to the antibacterial activity. It should be mentioned that chlorine and methoxide derivatives increased the antibacterial properties of the compound. In addition, the substitution of barbituric acid with thiobarbituric acid or dimethylbarbituric acid decreased the antibacterial properties of pyrazolopyranopyrimidine with similar derivatives. The antibacterial activity of the dihydropyrano[2,3-*c*]pyrazole derivatives against Gram-positive and Gram-negative bacteria was evaluated as shown in Table 5, wherein no significant effect was observed against the tested strains. The substitution of barbituric acid with malononitrile resulted in the elimination of the antibacterial effect observed in the targeted derivatives (Fig. 10).

**Table 4:** Antibacterial activity results for compounds (6a–6l)

Minimum inhibitory concentration (MIC = μg mL^−1^)													
Compound		6a	6b	6c	6d	6e	6f	6g	6h	6i	6j	6k	6l
Test organism	*S. aureus*	625	156.2	156.2	>2500	312.5	312.5	625	>2500	1250	>2500	312.5	>2500
	*E. coli*	>2500	1250	>2500	>2500	1250	1250	1250	1250	1250	>2500	1250	>2500

**Table 5:** Antibacterial activity results for compounds (7a–7l)

Minimum inhibitory concentration (MIC = μg mL^−1^)													
Compound		7a	7b	7c	7d	7e	7f	7g	7h	7i	7j	7k	7l
Test organism	*S. aureus*	>2500	>2500	>2500	>2500	>2500	>2500	>2500	>2500	>2500	>2500	>2500	>2500
	*E. coli*	—	—	—	—	—	—	—	—	—	—	—	—

### 3.5. Catalyst recycling and reusing test

The model reaction was used to examine the recovery and recyclability of CoFe_2_O_4_@SiO_2_-EA-H_3_PO_4_ MNPs. Upon completion of the reaction, an external magnetic field was applied to facilitate the separation of the catalyst from the reaction mixture. The catalyst was subsequently washed with EtOH, dried, and reused in subsequent reactions.

**Fig. 11:**
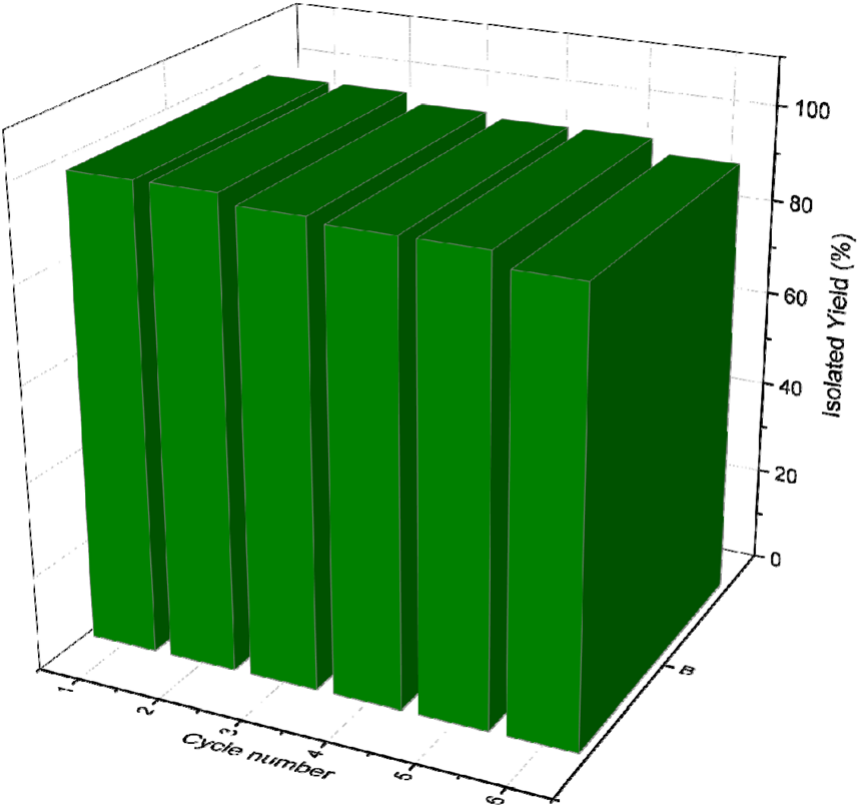
Recyclability of CoFe_2_O_4_@SiO_2_-EA-H_3_PO_4_ for the synthesis of pyrazolopyranopyrimidine derivatives.

**Fig. 12:**
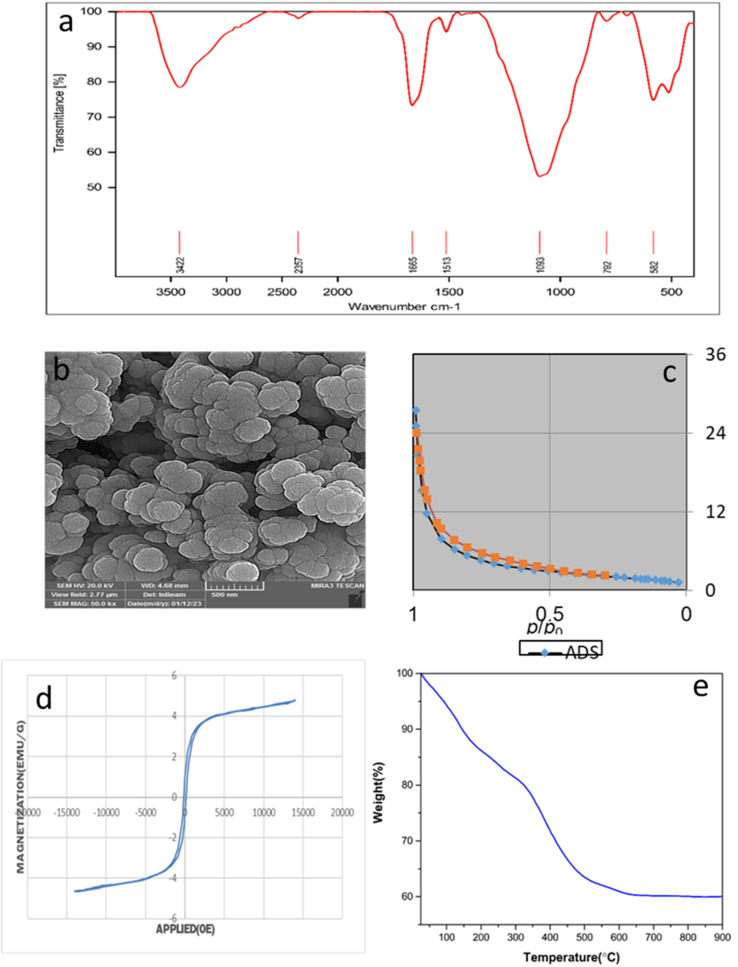
(a) FT-IR, (b) SEM, (c) BET, (d) VSM TGA and (e) TGA of the CoFe_2_O_4_@SiO_2_-EA-H_3_PO_4_ nanocatalyst after 6 runs.

This catalyst showed little deactivation after six uses, which is an important aspect of green chemistry. The reactions were conducted under optimal conditions, the results of which are presented in Fig. 11. To evaluate the durability and reusability potential of the CoFe_2_O_4_@SiO_2_-EA-H_3_PO_4_ particles, some specific analyses were used applying FT-IR, SEM, VSM, TGA, and BET (Fig. 12).

The results of the various tests conducted on the recycled catalyst confirm that the catalytic components attached to the CoFe_2_O_4_ support and its morphology remain stable throughout the reaction and recycling process. The N_2_ adsorption/desorption isotherms of the regenerated catalyst reveal a BET specific surface area (SSA) and total pore volume (TPV) of approximately SSA = 96.64 m^2^ g^−1^ and 1.98 cm^3^ g^−1^, respectively. These values indicate a minimal decrease as compared to the fresh catalyst, affirming its stability throughout the catalytic process.

### 3.6. Hot-filtration and leaching test

In order to investigate the heterogeneity of the catalyst in the reaction medium, a hot filtration test was performed on the model reaction. The catalyst was separated using a magnet at the midway point of the reaction and, then, the resulting filtrate solution was employed to resume the process. Following the hot filtration experiment, no change in the yield of the product was witnessed, which is comparable to the catalyst-free reaction conditions. In addition, ICP-OES analysis of the filtrate solution showed that there was almost no phosphor leaching into the reaction medium. These results indicate the heterogeneous nature and no leakage of the catalyst in the course of the reaction.

### 3.7. Comparison

In order to investigate the activity of the CoFe_2_O_4_@SiO_2_-EA-H_3_PO_4_ nanocatalyst in synthesizing pyrazolopyranopyrimidine, this system was compared to previous studies concerning the model reaction (product 6b), the results of which are illustrated in Table 6. These results demonstrate that the utilization of the CoFe_2_O_4_@SiO_2_-EA-H_3_PO_4_ nanocatalyst for the synthesis of pyrazolopyranopyrimidine offers advantages such as high yield, reduced reaction time, and lower temperature as compared to other catalysts.

**Table 6:** Comparison of the activity of the CoFe_2_O_4_@SiO_2_-EA-H_3_PO_4_ nanocatalyst with other catalysts for the synthesis of pyrazolopyranopyrimidine

Entry	Catalyst	Conditions	Time (min)	Yield (%)
1	TiO_2_ NWs	10 mol%, H_2_O/EtOH, reflux	60	95 (ref. 31)
2	SBA-Pr-SO_3_H	H_2_O, reflux	10	92 (ref. 32)
3	Oleic acid	EtOH/reflux	15	78 (ref. 33)
4	Meglumine	0.1 mmol, H_2_O, rt	15	92 (ref. 34)
5	ABCO	20 mol%, H_2_O, reflux	20	99 (ref. 35)
6	HPA-FHNTs	30 mg, H_2_O, reflux	35	95 (ref. 36)
7	MWCNTs/CO_2_H	5 mg, H_2_O/EtOH, reflux	80	92 (ref. 10)
8	Choline chloride/urea	20 mol%, EtOH, reflux	60	78 (ref. 37)
9	CoFe_2_O_4_@SiO_2_-PA-CCGuanidine	30 mg, H_2_O, rt	15	97 (ref. 10)
10	MWCNTs/guanidine/Ni(ii)	5 mg, EtOH, ultrasound	5	95 (ref. 37)
11	CoFe_2_O_4_@SiO_2_-EA-H_3_PO_4_	20 mg, water, rt	5	98 [this work]

## 4 Conclusion

In conclusion, this study demonstrated that the CoFe_2_O_4_@SiO_2_-EA-H_3_PO_4_ nanocomposite is an efficient and highly reusable nanomagnetic catalyst for the synthesis of pyrazolopyranopyrimidines and dihydropyrano[2,3-*c*]pyrazole *via* a one-pot four-component reaction under mild conditions with minimal energy consumption. Some of the important advantages of this catalytic system are as follows: high reusability of the nanocatalyst, high purity and yield of the products, environmentally friendly conditions and simple operation.

## Author contributions

Ali Mirzaie (the PhD student) performed the practical laboratory work as part of his PhD thesis. Lotfi Shiri (Supervisor, PhD) designed and coordinated the study, devised the concept, edited the final version of the manuscript, and submitted the manuscript for publication. Mosstafa Kazemi (Adviser, PhD) edited the final version of the manuscript draft. Nourkhoda Sadeghifard performed the antibacterial activity examination. Vahab Hassan Kaviar performed the antibacterial activity examination.

## Conflicts of interest

The authors declare no conflicts of interest.

## Supplementary Material

NA-006-D3NA00900A-s001
