# Supplementary material for: Nanomagnetic CoFe2O4@SiO2-EA-H3PO4 as a zwitterionic catalyst for the synthesis of bioactive pyrazolopyranopyrimidines and dihydropyrano[2,3-c]pyrazoles
Source: Nanoscale Adv. 2024 Feb 5;6(4):1227–40. doi: 10.1039/d3na00900a (PMC10863708; doi:10.1039/d3na00900a)
Supplement: NA-006-D3NA00900A-s001 [file NA-006-D3NA00900A-s001.pdf]

Electronic Supplementary Material (ESI) for Nanoscale Advances.
This journal is © The Royal Society of Chemistry 2024

Nanomagnetic $CoFe_2O_4@SiO_2$-EA-H_3PO_4 as an efficient and reusable catalyst for synthesis of pyrazolopyranopyrimidines and dihydropyrano[2,3-c]pyrazoles and Investigation of Their Antibacterial activity

Ali Mirzaie [a], Lotfi Shiri [a]*, Mosstafa Kazemi [a], Nourkhoda Sadeghifard [b], Vahab Hassan Kaviar [b]

[a] Department of Chemistry, Faculty of Basic Sciences, Ilam University, P.O. Box 69315-516, Ilam, Iran.

[b] Clinical Microbiology Research Center, Ilam University of Medical Sciences, Ilam, Iran,

*correspondence to: Lotfi Shiri

Email: l.shiri@ilam.ac.ir

SPECTROSCOPIC DATA OF SELECTED COMPOUNDS

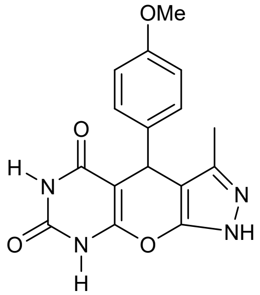

4-(4-methoxyphenyl)-3-methyl-4,8-dihydropyrazolo[4',3':5,6]pyrano[2,3-d]pyrimidine-5,7(1H,6H)-dione

IR (KBr) n = 3430, 2880, 2827, 1702, 1464, 1359, 1040; 1 H NMR (DMSO-d6, 250 MHz): 11.03 (s, 1H), 10.22 – 9.95 (m, 2H), 6.92 (d, 2H), 6.75 (d, 2H), 5.34 (s, 1H), 3.65 (s, 3H), 2.17 (s, 3H). C NMR (DMSO-d6, 62.5 MHz): 166.00, 161.03, 157.55, 151.10, 143.76, 134.84, 132.27 , 128.11, 128.10, 113.65, 106.53, 55.36, 10.45.

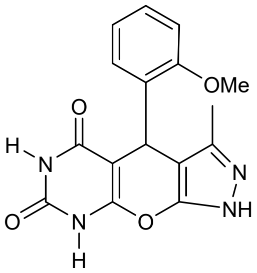

4-(2-methoxyphenyl)-3-methyl-4,8-dihydropyrazolo[4',3':5,6]pyrano[2,3-d]pyrimidine-5,7(1H,6H)-dione

IR (KBr) n = 3453, 2894, 2831, 1698, 1484, 1388, 1040; 1 H NMR (DMSO-d6, 250 MHz): 11.10 (s, 1H), 10.07 (m, 2H), 6.79-7.30 (m, 4H), 5.58 (s, 1H), 3.64 (s, 3H), 2.20 (s, 3H). 13C NMR (DMSO-d6, 62.5 MHz): 166.00, 160.72, 156.38, 152.44, 146.43, 139.33, 126.26 , 124.42, 122.75, 115.31, 106.36, 51.03, 6.00.

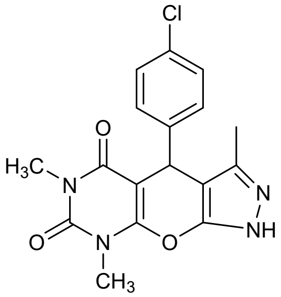

4-(4-chlorophenyl)-3,6,8-trimethyl-4,8-dihydropyrazolo[4',3':5,6]pyrano[2,3-d]pyrimidine-5,7(1H,6H)-dione

IR (KBr) n = 3390, 3189, 3062, 2971, 1688, 1559, 1498, 1416, 1264, 1133; H NMR (DMSO-d6, 250 MHz): δ 13.95 (s, 1H), 7.41 – 6.96 (d-d, 4H), 4.43 (s, 1H), 3.40 (s, 3H), 1.98 (s, 3H), 1.68 (s, 3H). C NMR (DMSO-d6, 62.5 MHz): 178.86, 160.28, 158.08, 154.89, 140.59, 137.46, 134.35, 129.77, 127.63, 127.58, 101.47, 55.45, 12.66, 9.92.

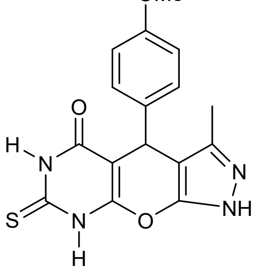

4-(4-methoxyphenyl)-3-methyl-7-thioxo-4,6,7,8-tetrahydropyrazolo[4',3':5,6]pyrano[2,3-d]pyrimidin-5(1H)-one

IR (KBr) n = 3230, 2997, 2827, 1620, 1518, 1404, 1173; H NMR (DMSO-d6, 250 MHz): 11.44 (s, 2H), 6.90 (d, 2H), 6.73 (d, 2H), 5.35 (s, 1H), 3.66 (s, 3H), 2.21 (s, 3H).C NMR (DMSO-d6, 62.5 MHz): 173.37, 163.73, 159.72, 157.10, 143.83, 134.14, 133.27 , 128.11, 128.05, 113.75, 106.03, 96.62, , 55.38 , 30.25 , 10.45

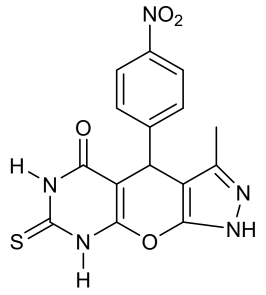

3-methyl-4-(4-nitrophenyl)-7-thioxo-4,6,7,8-tetrahydropyrazolo[4',3':5,6]pyrano[2,3-d]pyrimidin-5(1H)-one

IR (KBr) n = 3429, 2976, 2879, 16188, 1527, 1433, 1310, 1169, 1116; H NMR (DMSO-d6, 250 MHz): δ 11.49 (d, 2H), 8.68 (s, 1H), 7.37 – 6.96 (d-d, 4H), 5.35 (s, 1H), 2.22 (s, 3H).
13C NMR (DMSO-d6, 62.5 MHz): 168.99, 159.05, 154.87, 139.55, 137.18, 127.85, 126.55, 124.80, 114.39, 91.52, 5.75.

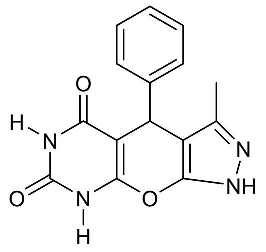

3-methyl-4-phenyl-4,8-dihydropyrazolo[4',3':5,6]pyrano[2,3-d]pyrimidine-5,7(1H,6H)-dione

IR (KBr): 3476, 3264, 3116, 2885, , 2257, 1650, 1600, 1520, 1452, 1352, 1106cm^{-}; ^{1}H NMR (250 MHz, DMSO) δ 11.11 (s, 1H), 10.09 – 10.03 (d, 2H), 7.18-7.02 (m, 5H), 5.39 (s, 1H), 2.18 (s, 3H).

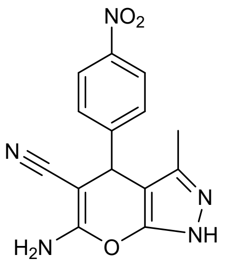

6-Amino-4-(4-Nitrophenyl)-3-methyl-2,4-dihydropyrano[2,3-c] pyrazole-5-carbonitrile :
IR (KBr: υ/cm 13): 3476, 3228, 2196, 1650, 1600 cm^{-1} ; ^{1}H NMR (250 MHz, DMSO) δ 12.19 (s, 1H), 8.18 (d, J = 8.3 Hz, 2H), 7.44 (d, J = 8.4 Hz, 2H), 7.04 (s, 2H), 4.81 (s, 1H), 1.77 (s, 3H). C NMR (125 MHz, DMSO-d6); δC (ppm) = 161.54, 155.09, 152.51, 146.80, 136.29, 129.24, 129.24, 127.05, 124.30, 120.88, 96.95, 56.33, 36.30, 10.4.

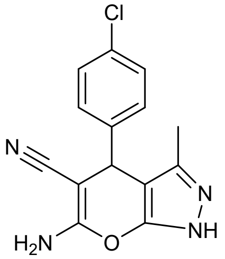

6-Amino-4-(4-chlorophenyl)-3-methyl-2,4-dihydropyrano[2,3-c] pyrazole-5-carbonitrile :
IR (KBr) n = 3390, 3189, 3062, 2971, 1688, 1559, 1498, 1416, 1264, 1133; H NMR (DMSO-d6, 250 MHz): δ 13.95 (s, 1H), 7.41 – 6.96 (d-d, 4H), 4.43 (s, 1H), 3.40 (s, 3H), 1.98 (s, 3H), 1.68 (s, 3H). C NMR (DMSO-d6, 62.5 MHz): 178.86, 160.28, 158.08, 154.89, 140.59, 137.46, 134.35, 129.77, 127.63, 127.58, 101.47, 55.45, 12.66, 9.92.

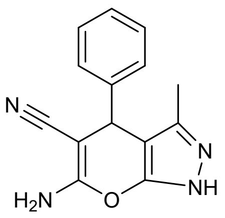

6-amino-3-methyl-4-phenyl-1,4-dihydropyrano[2,3-c]pyrazole-5-carbonitrile

IR (KBr): 3377, 3029, 2189, 1645, 1526 cm^{-1} ; ^{1}H NMR (250 MHz, DMSO) δ 12.08 (s, 1H), 7.64 – 6.86 (m, 5H), 6.26 – 4.47 (s, 2H), 1.90 (s, 3H).

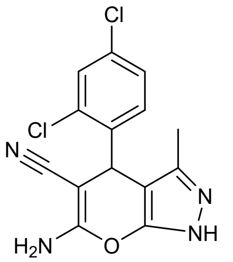

4-(2,4-chlorophenyl)-3,6,8-trimethyl-4,8-dihydropyrazolo[4',3':5,6]pyrano[2,3-d]pyrimidine-5,7(1H,6H)-dione

IR (KBr) n = 3433, 2923, 2358, 1730, 1583, 1464, 1383, 1203; ^{1}H NMR (250 MHz, DMSO) δ 8.88 (s, 1H), 8.13 (s, 1H), 7.77 (s, 1H), 7.55 (s, 2H), 4.52 (s, 1H), 1.92 (s, 3H).

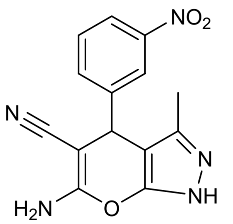

6-amino-3-methyl-4-(3-nitrophenyl)-1,4-dihydropyrano[2,3-c]pyrazole-5-carbonitrile

IR (KBr: υ/cm 13): 3473, 3226, 2193, 1653, 1526 cm $^{-1}$; ^{1}H NMR (250 MHz, DMSO) δ 12.20 (s, 1H), 7.04 – 8.11 (m, 4H), 8.80 (s, 2H), 4.86 (s, 1H), 1.79 (s, 3H). ^{13} C NMR (125 MHz, DMSO-d6); δC (ppm) = 156.96, 150.49, 143.70, 142.58, 131.75, 130.14, 125.99, 117.67, 116.29, 92.42, 52.01, 5.51.

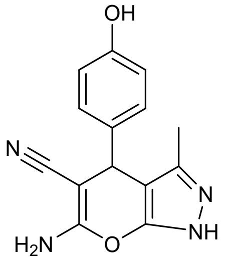

6-amino-4-(4-hydroxyphenyl)-3-methyl-1,4-dihydropyrano[2,3-c]pyrazole-5-carbonitrile

IR (KBr): 3395, 3137, 2176, 1647, 1604 cm $^{-1}$; ^{1}H NMR (250 MHz, DMSO) δ 12.04 (s, 1H), 9.29 (s,1H), 6.92 (s, 2H), 6.95 (m, 2H), 6.88 (m, 2H), 4.41 (s, 1H), 1.76 (s, 3H).^{13} C NMR (125 MHz, DMSO-d6); δC (ppm) = 156.44, 151.83, 150.54, 131.36, 130.56, 124.23, 116.70, 110.92, 110.28, 93.86, 53.64, 5.54.

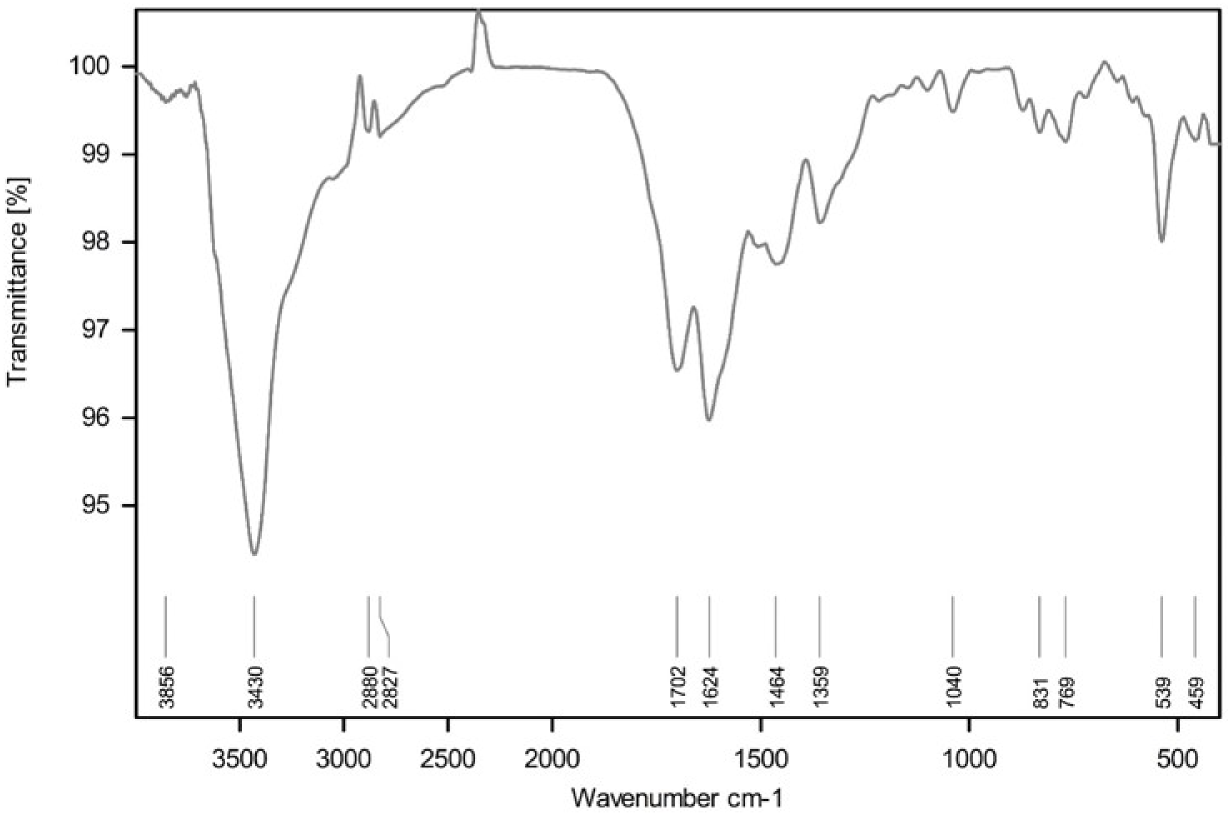

Figure S1. FT-IR of 4-(4-methoxyphenyl)-3-methyl-4,8-dihydropyrazolo[4',3':5,6]pyrano[2,3-d]pyrimidine-5,7(1H,6H)-dione in KBr.

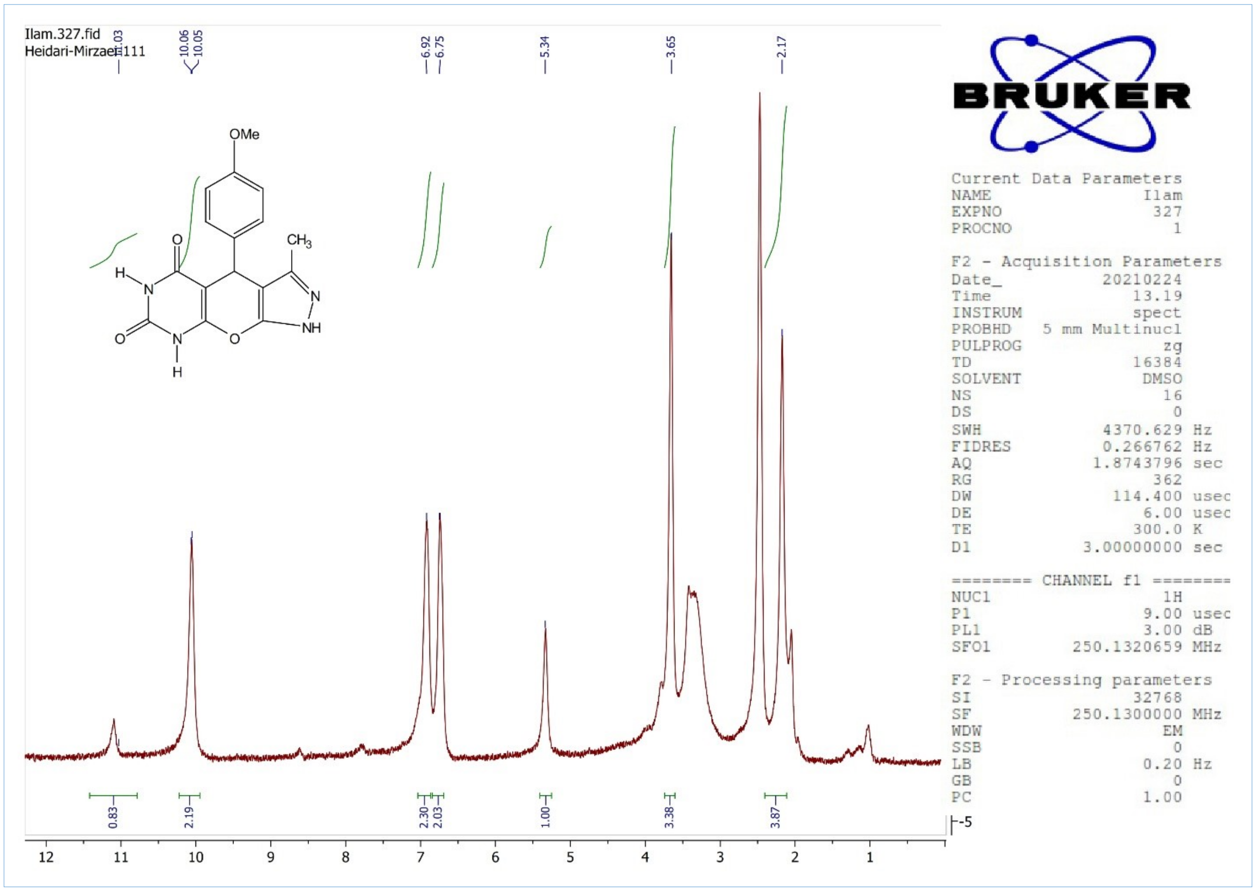

Figure S2. ^{1}H NMR of 4-(4-methoxyphenyl)-3-methyl-4,8-dihydropyrazolo[4',3':5,6]pyrano[2,3-d]pyrimidine-5,7(1H,6H)-dione in DMSO.

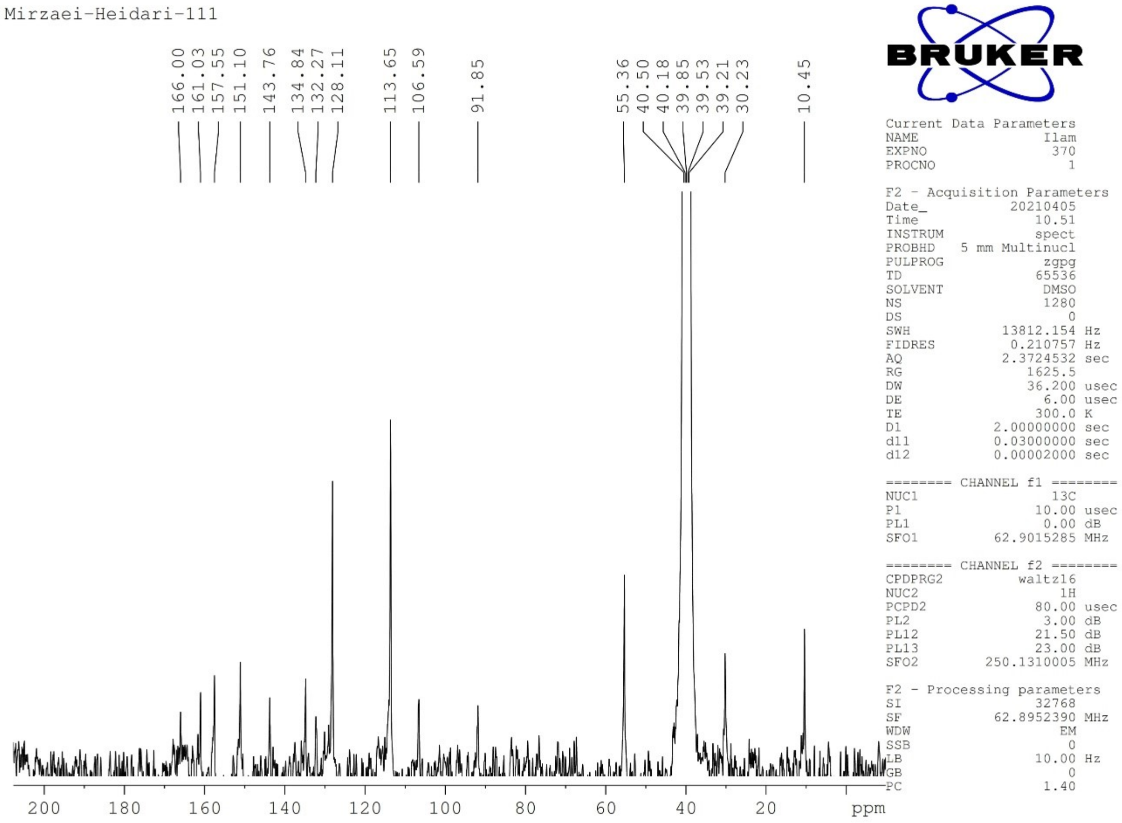

Figure S3. ^{13}C NMR of 4-(4-methoxyphenyl)-3-methyl-4,8-dihydropyrazolo[4',3':5,6]pyrano[2,3-d]pyrimidine-5,7(1H,6H)-dione in DMSO.

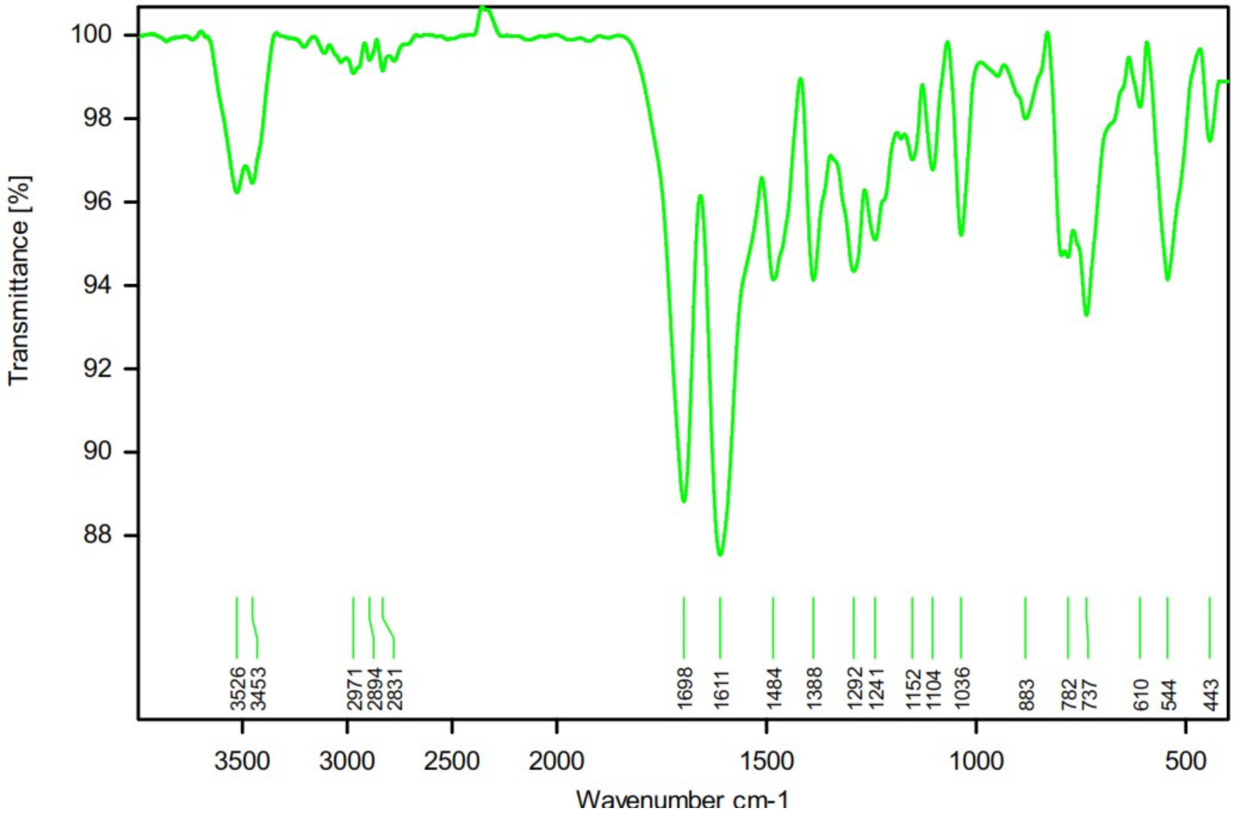

Figure S4. FT-IR of 4-(2-methoxyphenyl)-3-methyl-4,8-dihydropyrazolo[4',3':5,6]pyrano[2,3-d]pyrimidine-5,7(1H,6H)-dione in KBr.

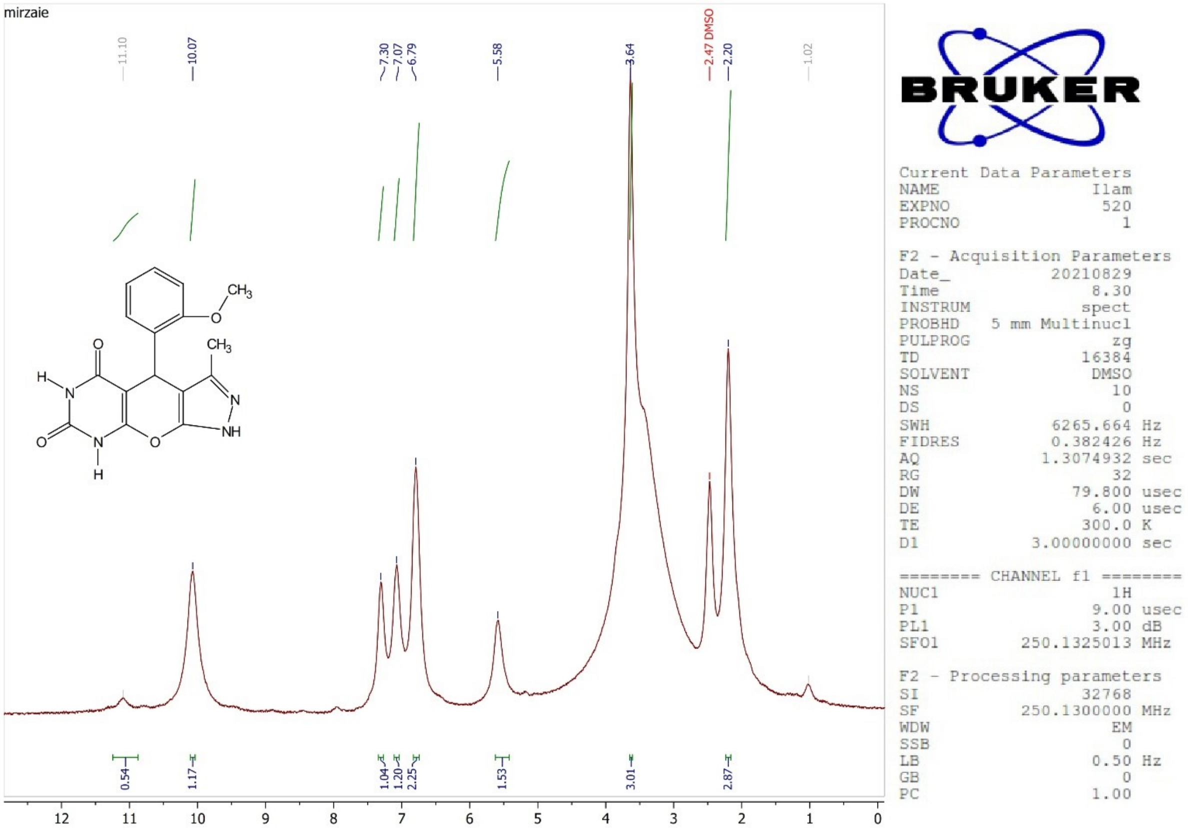

Figure S5. ^{1}H NMR of 4-(2-methoxyphenyl)-3-methyl-4,8-dihydropyrazolo[4',3':5,6]pyrano[2,3-d]pyrimidine-5,7(1H,6H)-dione in DMSO.

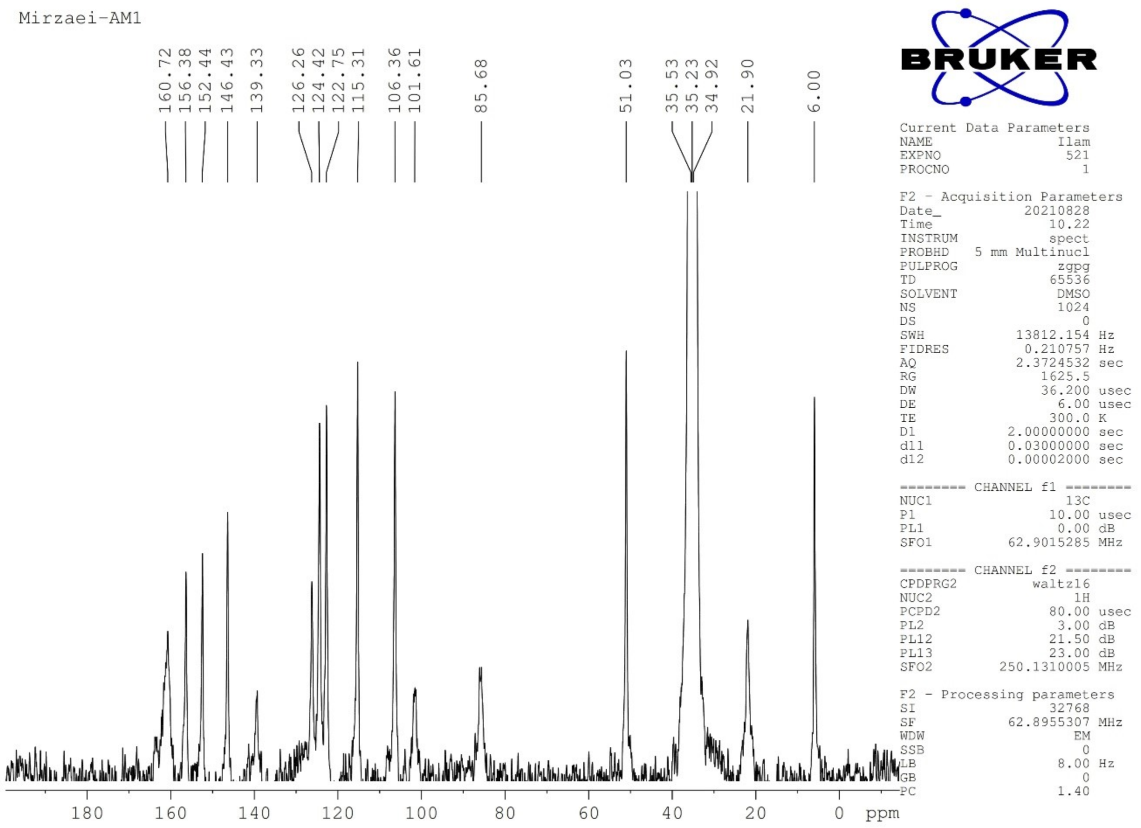

Figure S6. ^{13}C NMR of 4-(2-methoxyphenyl)-3-methyl-4,8-dihydropyrazolo[4',3':5,6]pyrano[2,3-d]pyrimidine-5,7(1H,6H)-dione in DMSO.

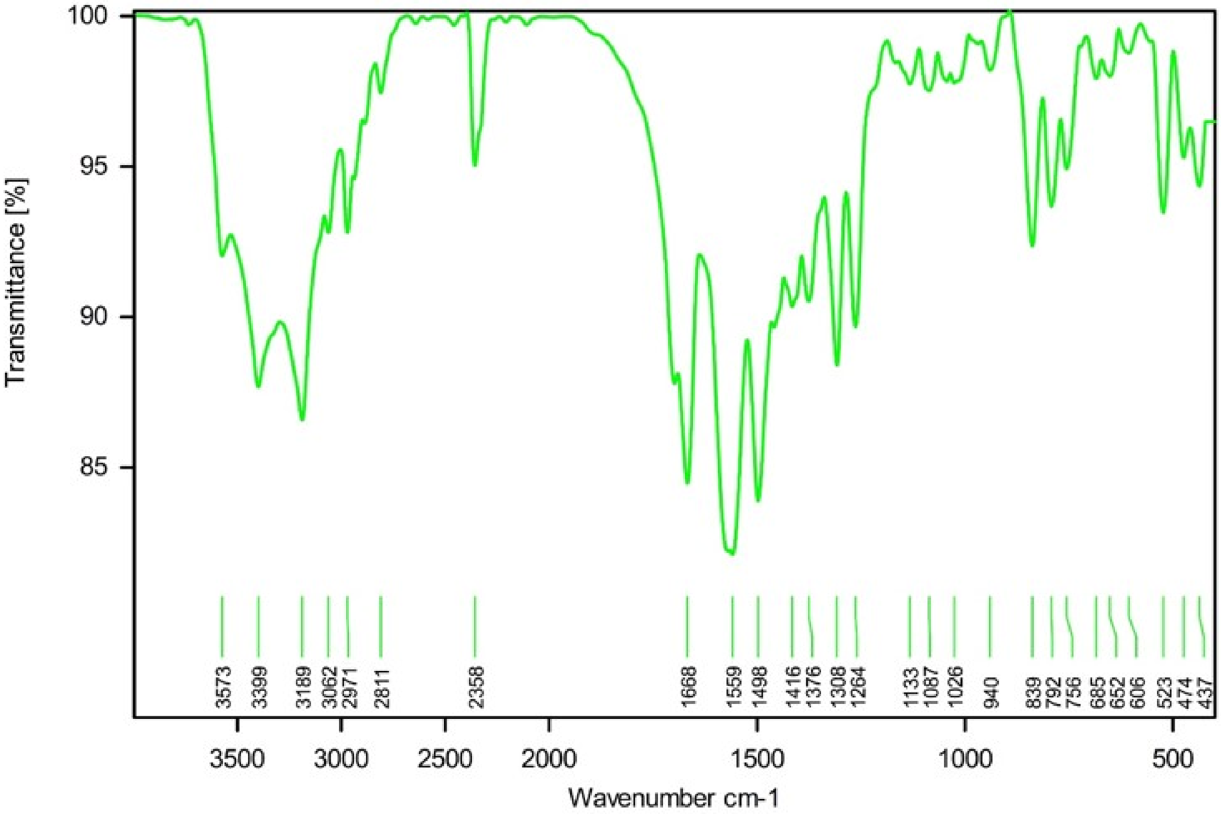

Figure S7. FT-IR of 4-(4-chlorophenyl)-3,6,8-trimethyl-4,8-dihydropyrazolo[4',3':5,6]pyrano[2,3-d]pyrimidine-5,7(1H,6H)-dione in KBr.

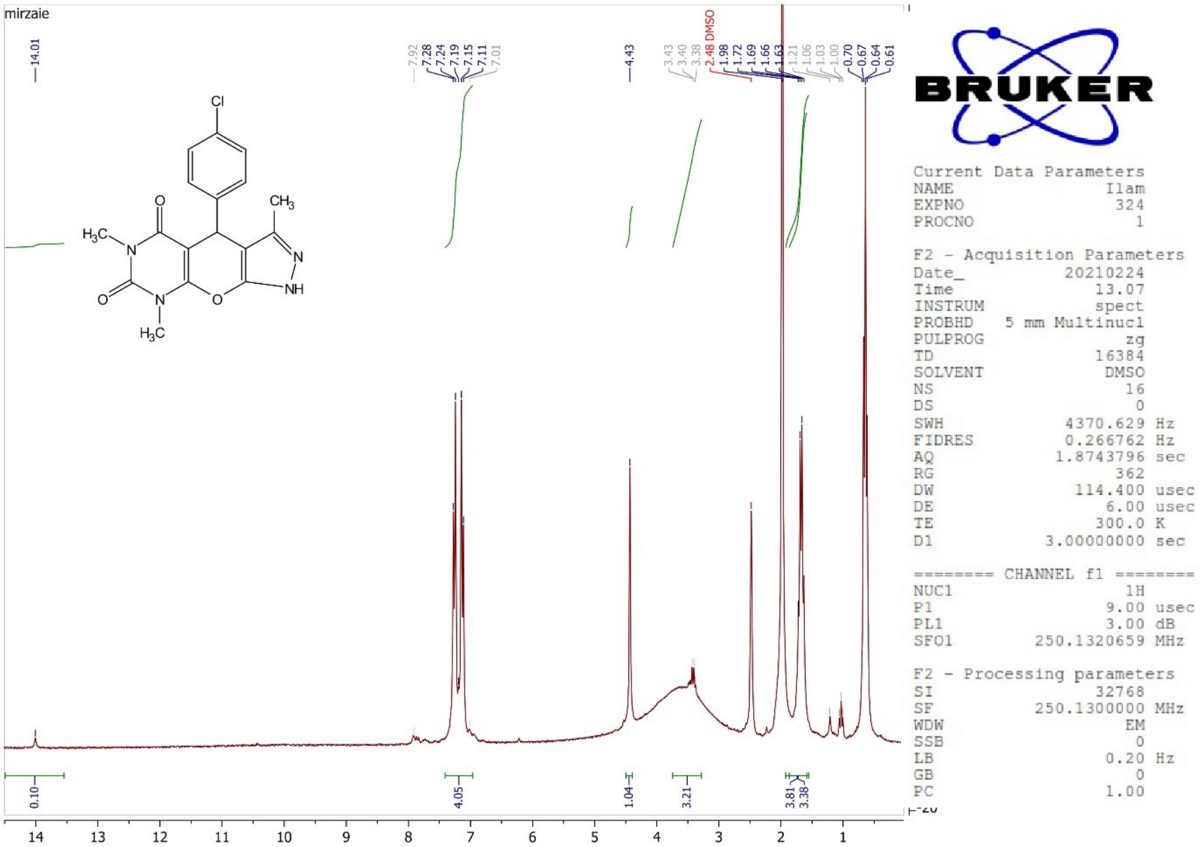

Figure S8. ^{1}H NMR of 4-(4-chlorophenyl)-3,6,8-trimethyl-4,8-dihydropyrazolo[4',3':5,6]pyrano[2,3-d]pyrimidine-5,7(1H,6H)-dione in DMSO.

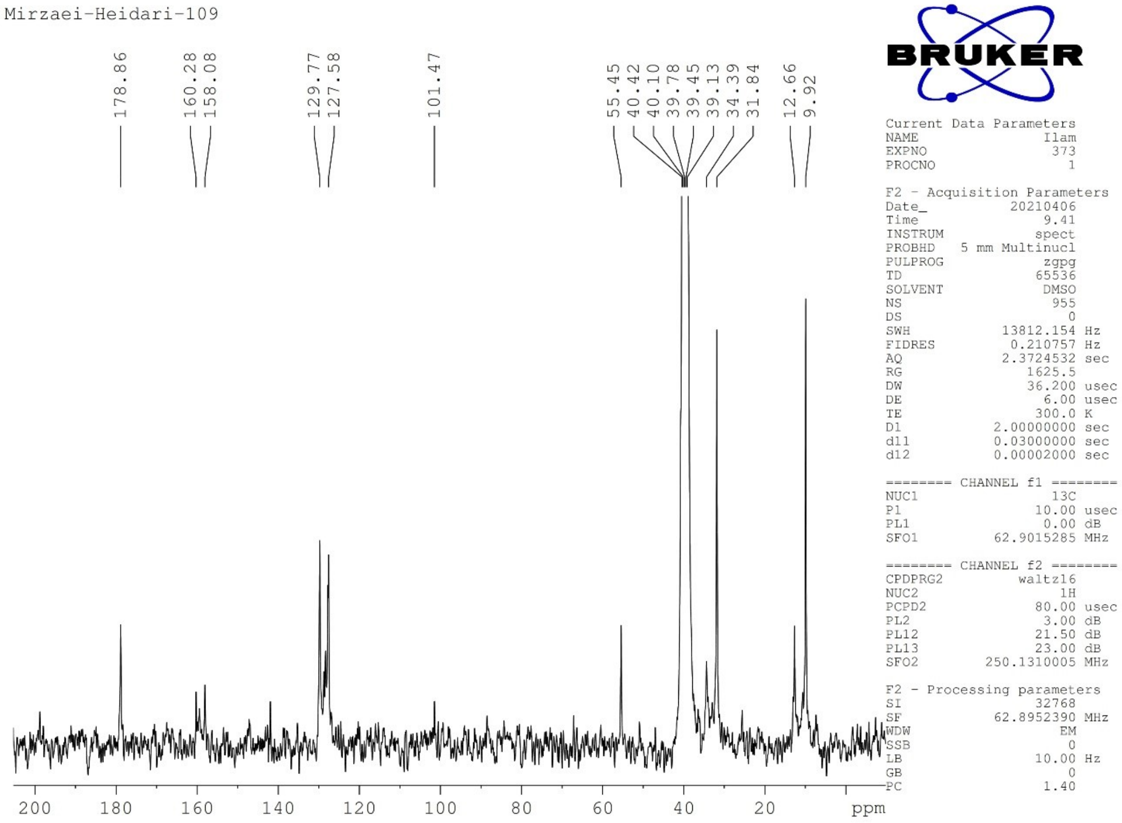

Figure S9. ^{13}C NMR of 4-(4-chlorophenyl)-3,6,8-trimethyl-4,8-dihydropyrazolo[4',3':5,6]pyrano[2,3-d]pyrimidine-5,7(1H,6H)-dione in DMSO.

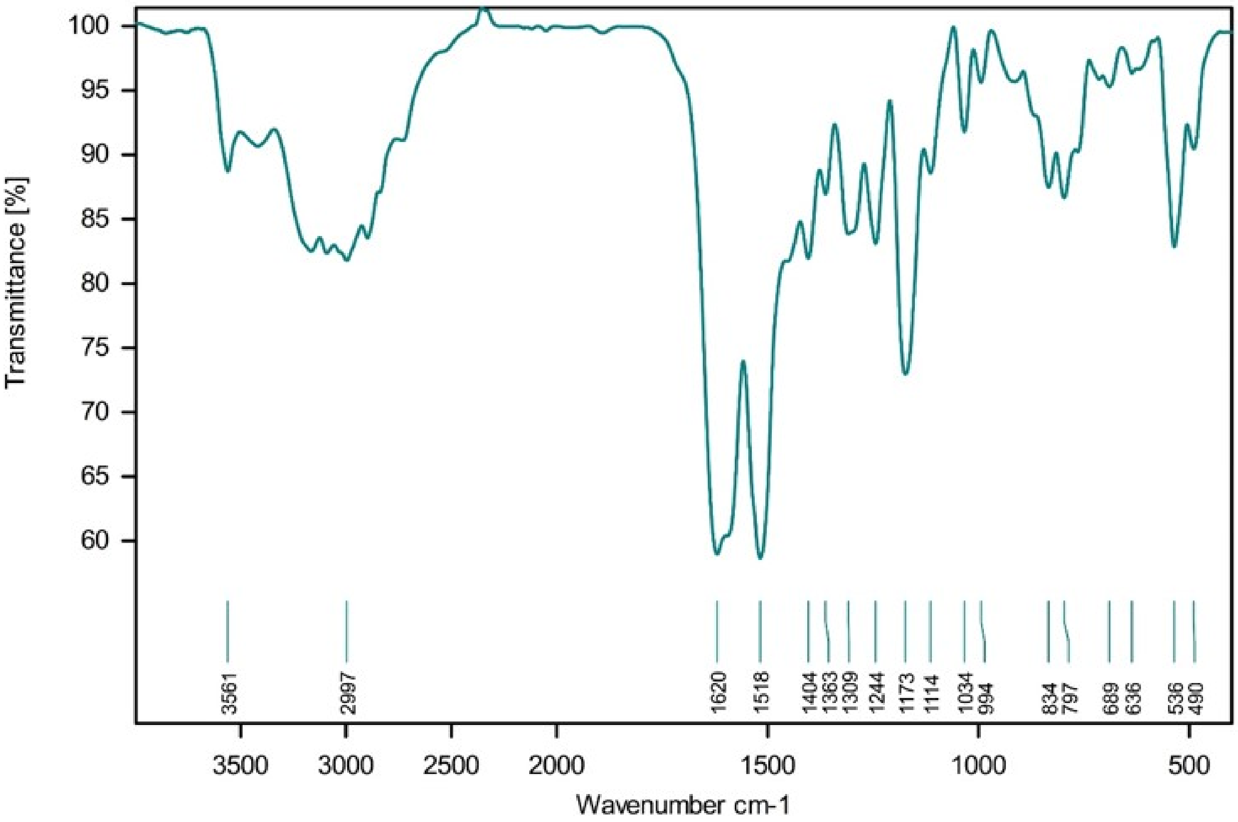

Figure S10.FTIR of 4(4methoxyphenyl)3methyl7thioxo4,6,7,8tetrahydropyrazolo[4',3':5,6]pyrano[2,3-d]pyrimidin-5(1H)-onein KBr.

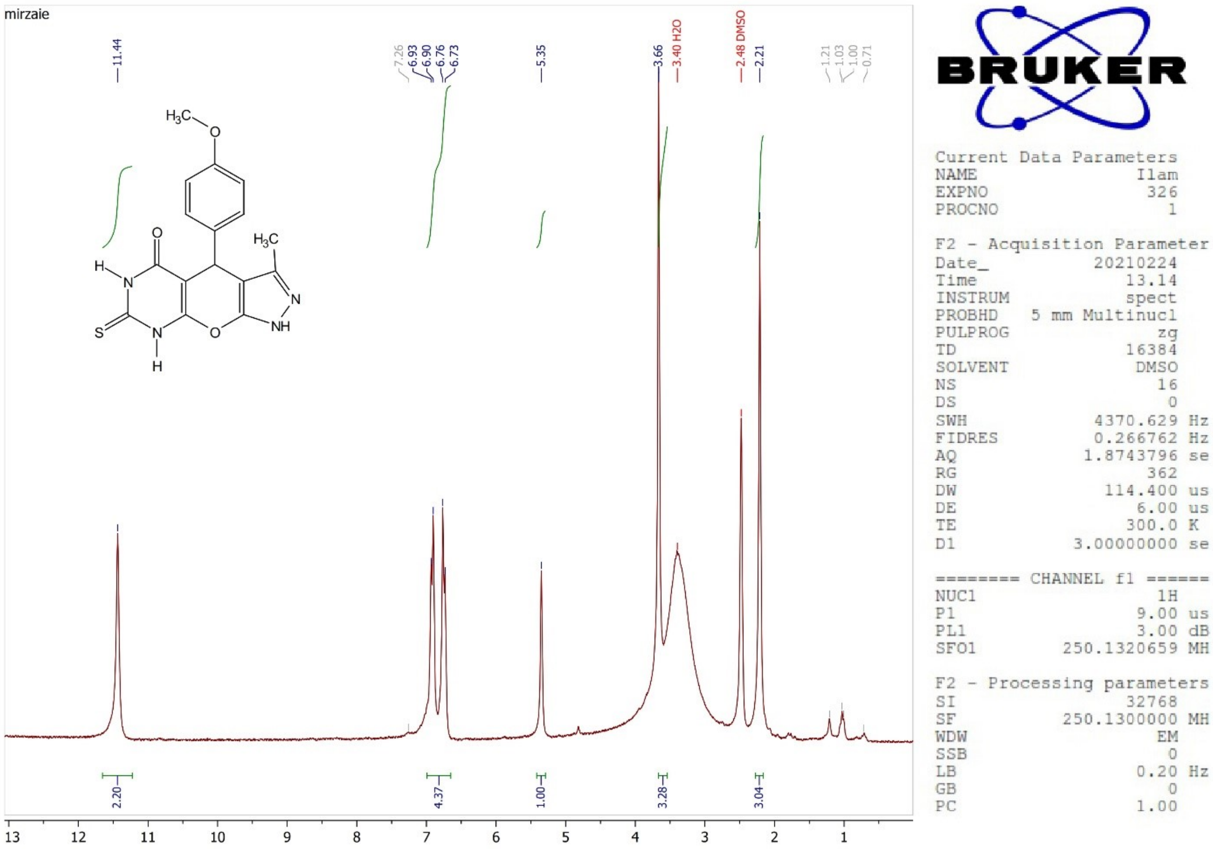

FigureS11.^{1}HNMR of 4(4methoxyphenyl)3methyl7thioxo4,6,7,8tetrahydropyrazolo[4',3':5,6]pyrano[2,3-d]pyrimidin-5(1H)-one in DMSO.

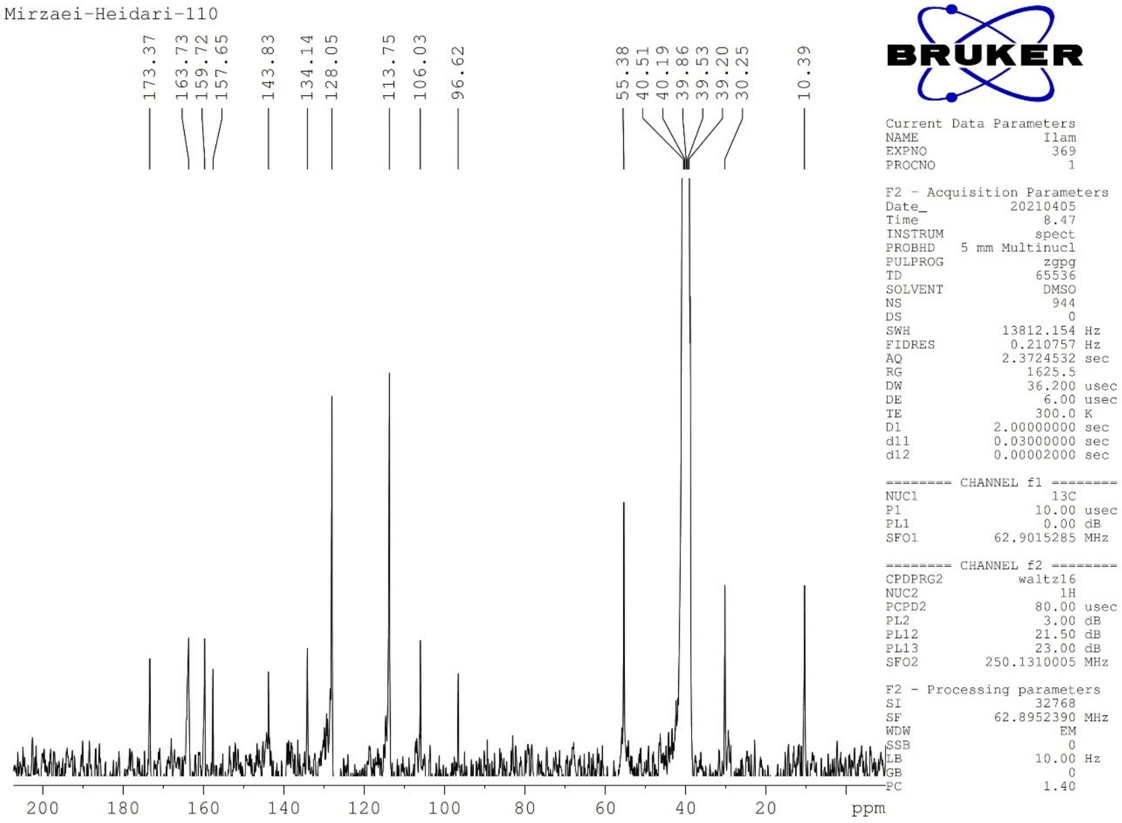

FigureS12. ^{13}CNMRof4(4methoxyphenyl)3methyl7thioxo4,6,7,8tetrahydropyrazolo[4',3':5,6]pyrano[2,3-d]pyrimidin-5(1H)-one in DMSO.

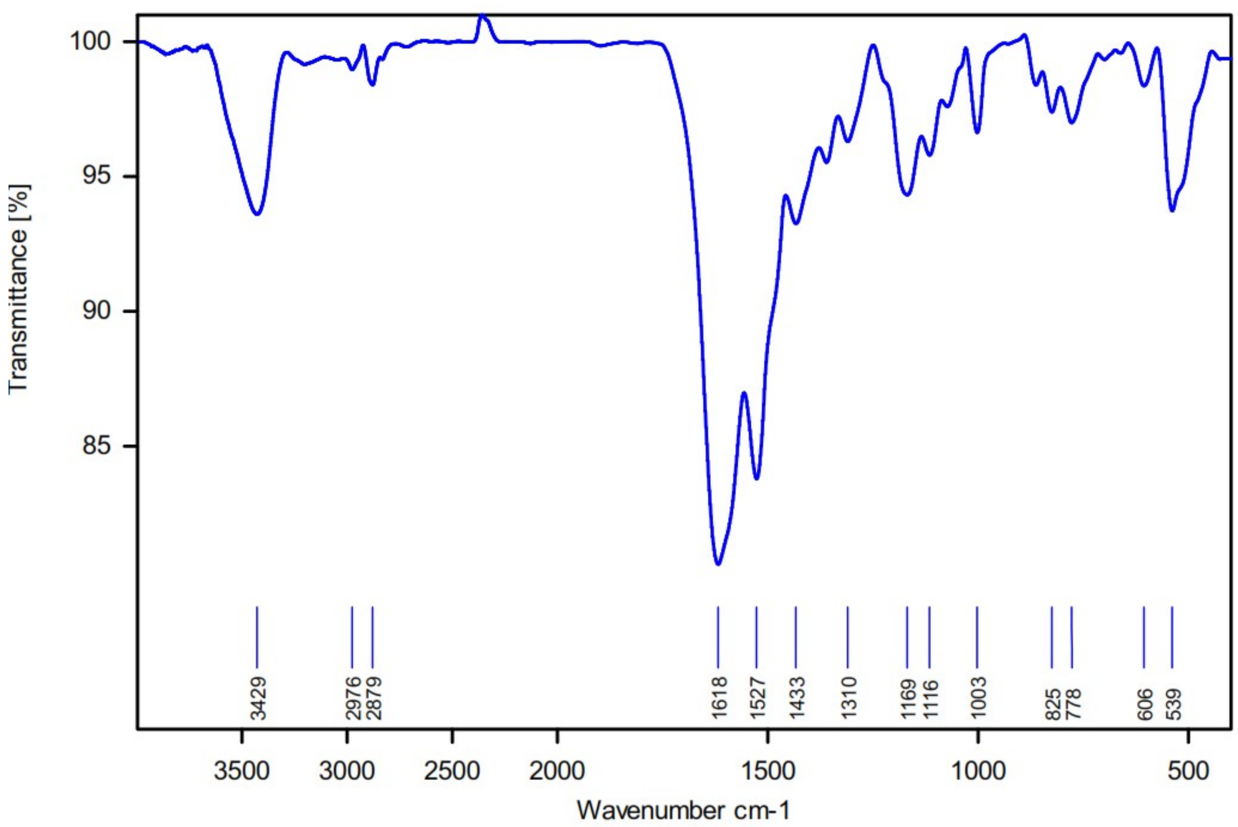

Figure S13.FTIR of 3-methyl-4-(4-nitrophenyl)-7-thioxo-4,6,7,8-tetrahydropyrazolo[4',3':5,6]pyrano[2,3-d]pyrimidin-5(1H)-one KBr.

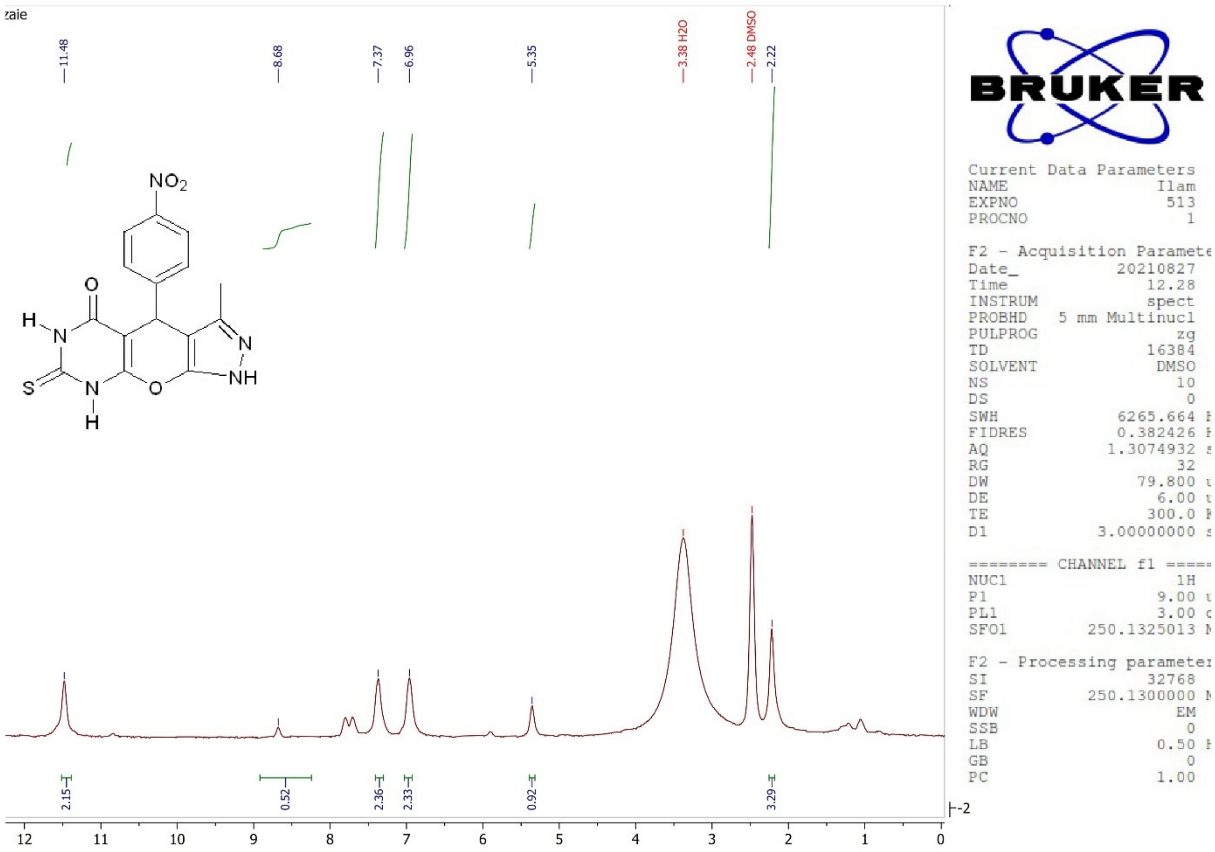

Figure S14. ^{1}H NMR of 3-methyl-4-(4-nitrophenyl)-7-thioxo-4,6,7,8-tetrahydropyrazolo[4',3':5,6]pyrano[2,3-d]pyrimidin-5(1H)-one in DMSO.

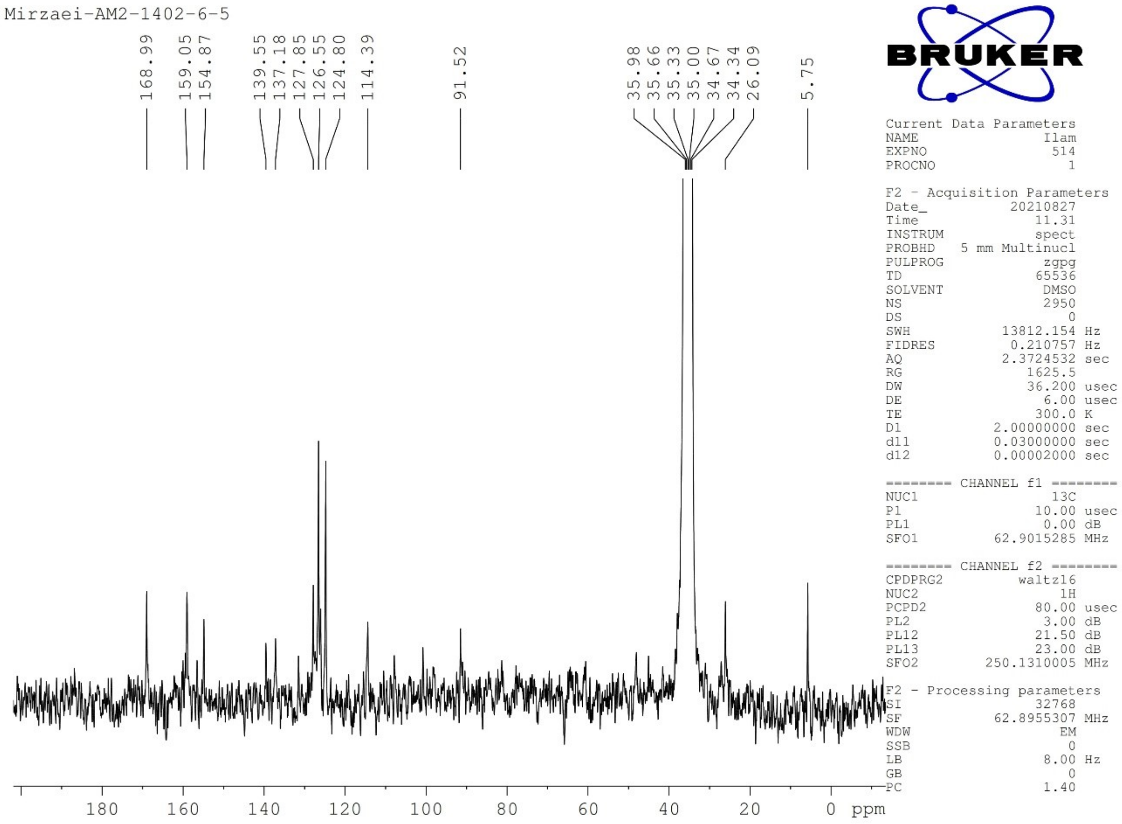

FigureS15.^{13}CNMRof 3-methyl-4-(4-nitrophenyl)-7-thioxo-4,6,7,8-tetrahydropyrazolo[4',3':5,6]pyrano[2,3-d]pyrimidin-5(1H)-one in DMSO.

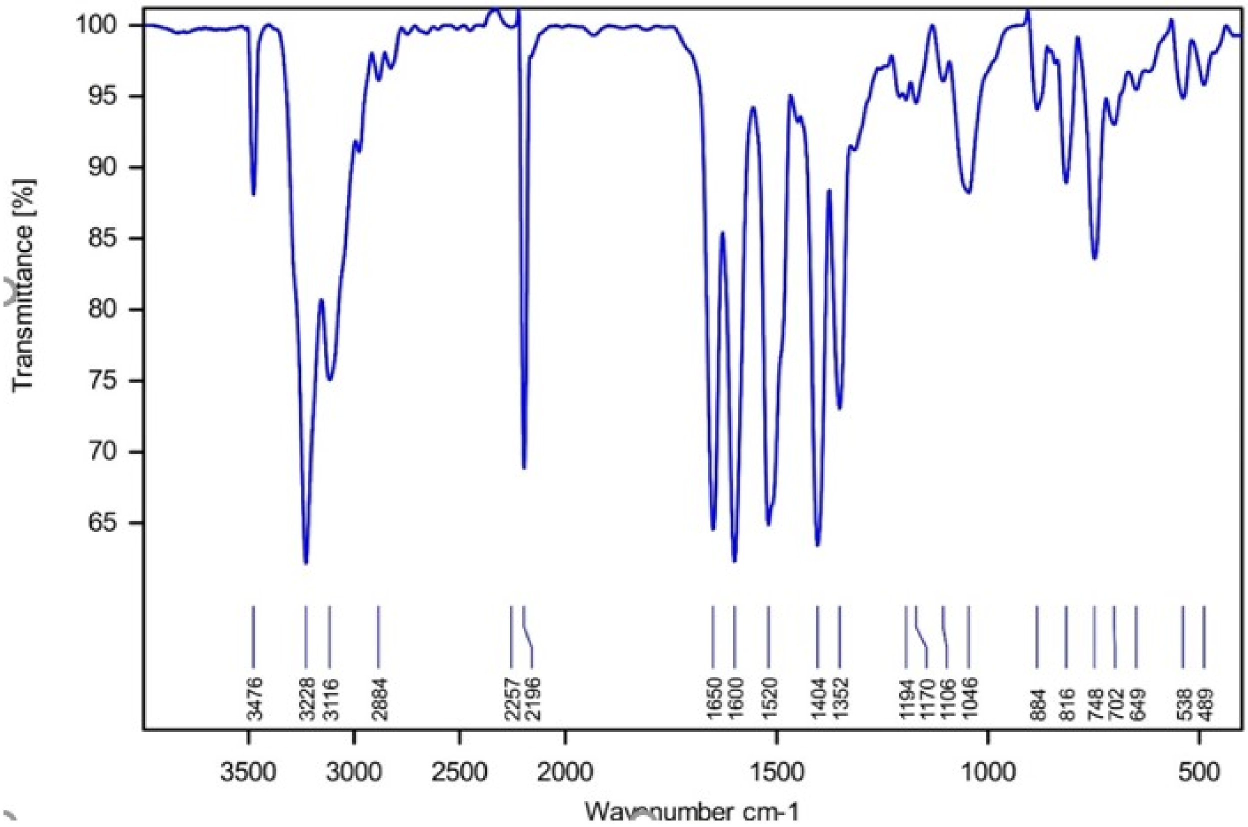

Figure S16. FT-IR of 3-methyl-4-phenyl-4,8-dihydropyrazolo[4',3':5,6]pyrano[2,3-d]pyrimidine-5,7(1H,6H)-dione in KBr.

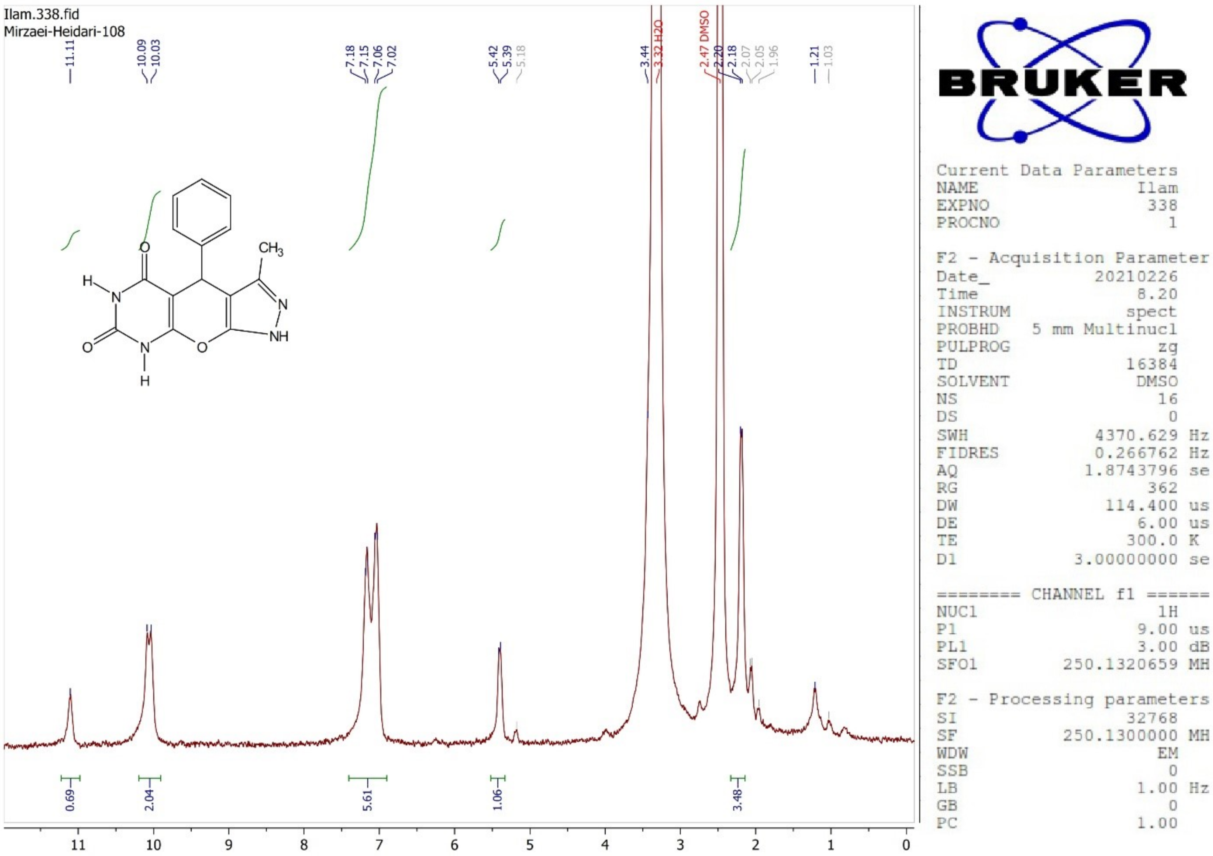

Figure S17. ^{1}H NMR of 3-methyl-4-phenyl-4,8-dihydropyrazolo[4',3':5,6]pyrano[2,3-d]pyrimidine-5,7(1H,6H)-dione in DMSO.

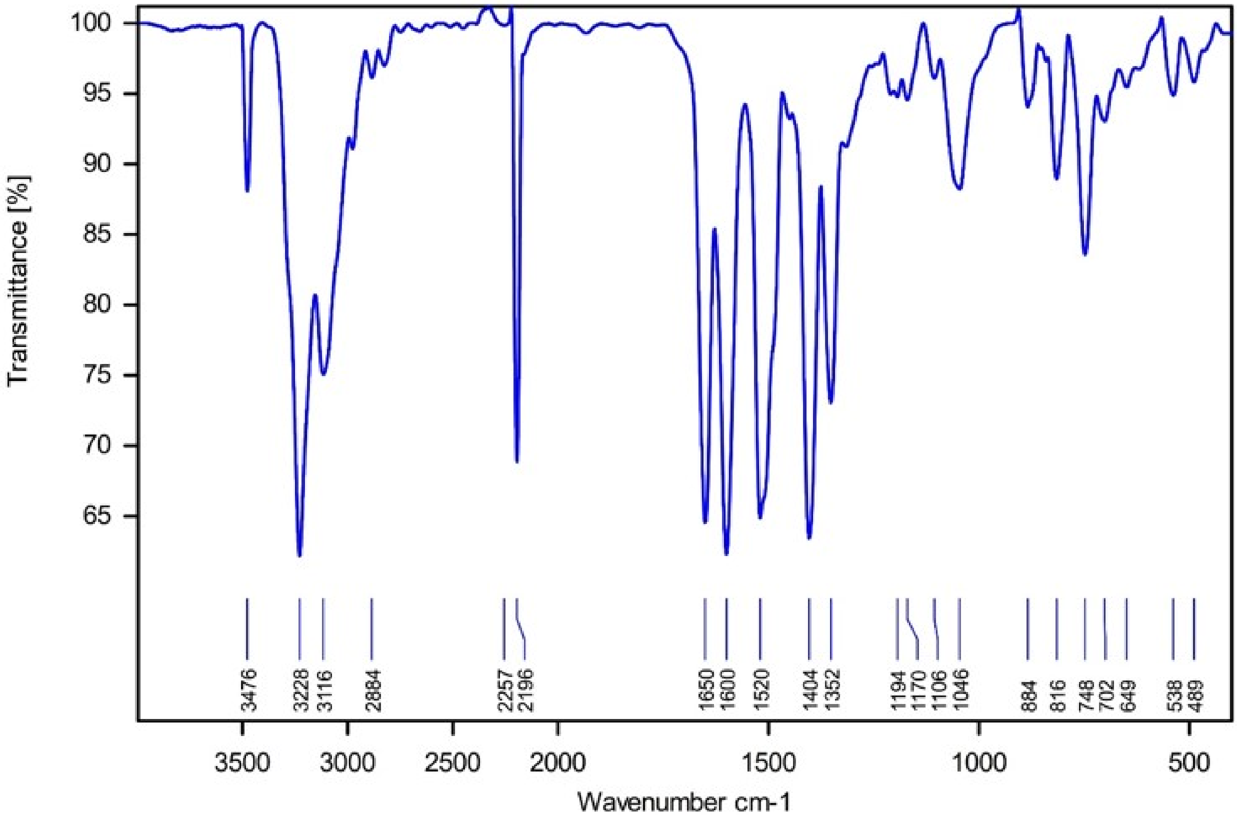

Figure S18. FT-IR of 6-Amino-4-(4-Nitrophenyl)-3-methyl-2,4-dihydropyrano[2,3-c] pyrazole-5-carbonitrile in KBr.

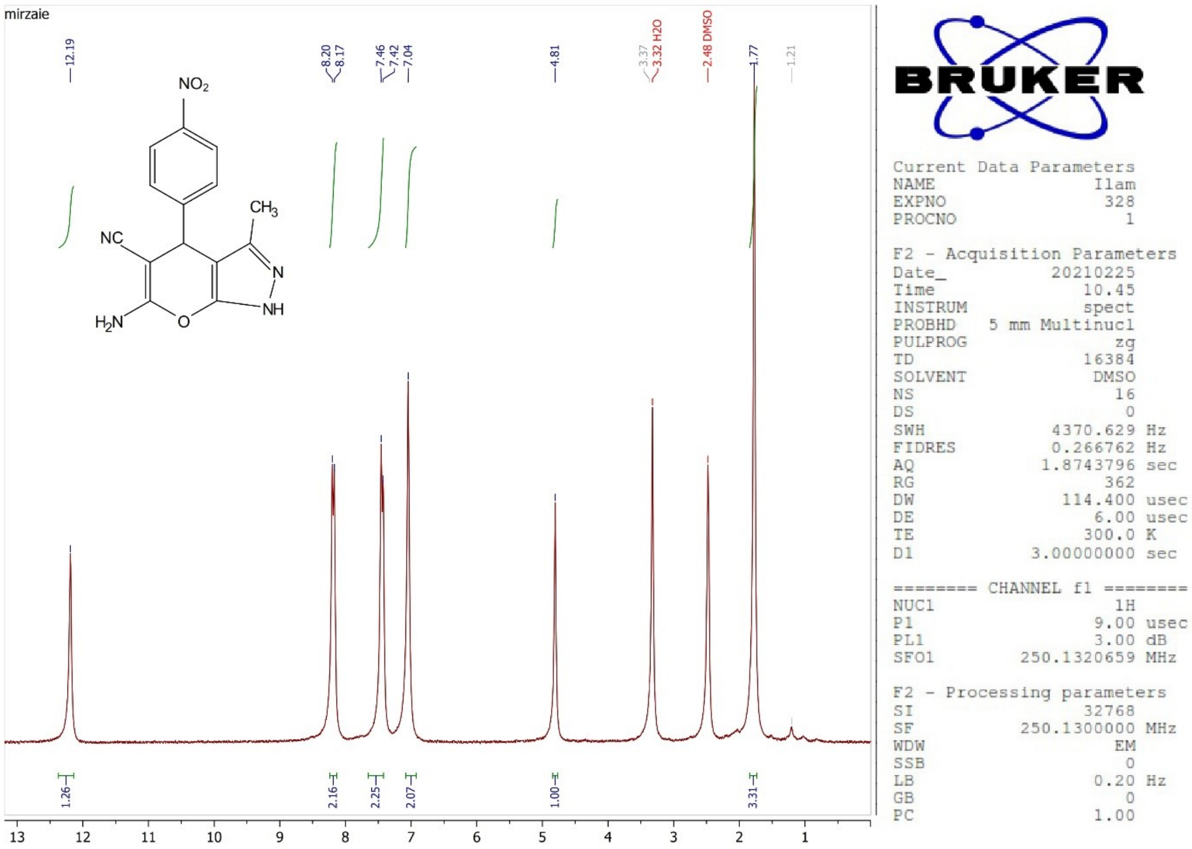

Figure S19. ^{1}H NMR of 6-Amino-4-(4-Nitrophenyl)-3-methyl-2,4-dihydropyrano[2,3-c] pyrazole-5-carbonitrile one in DMSO.

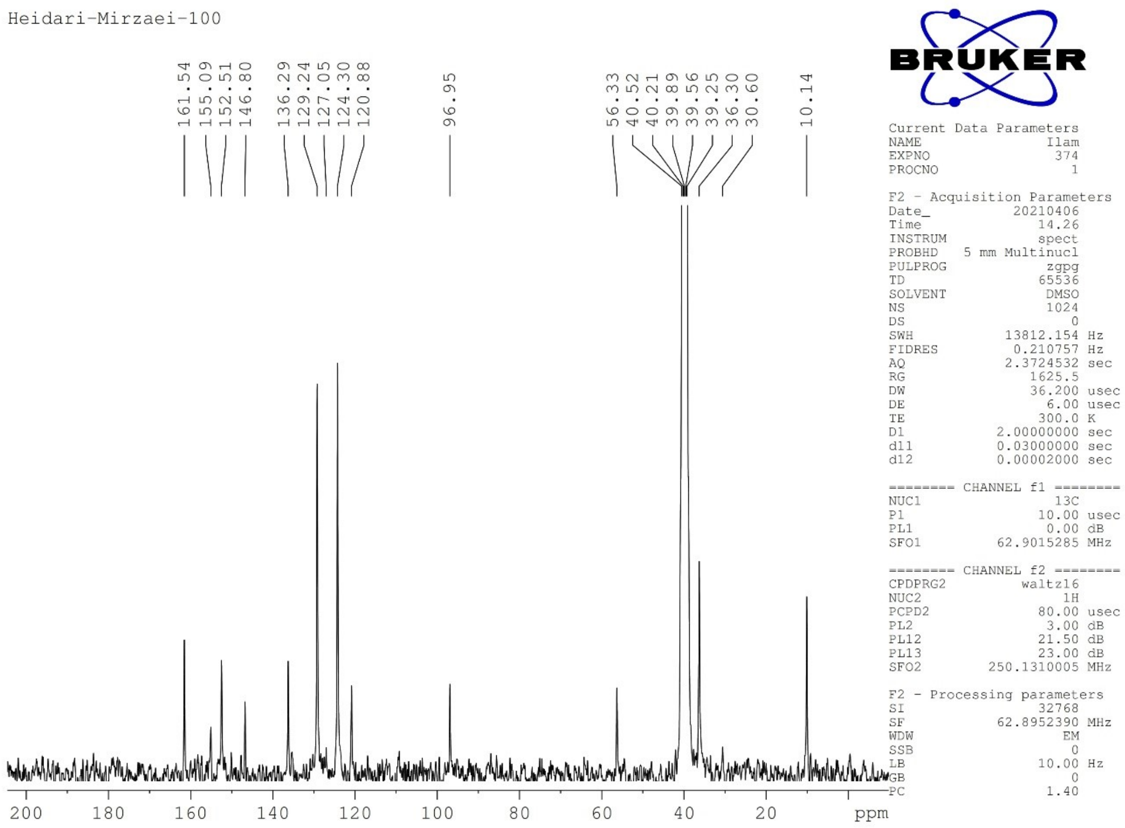

Figure S20. ^{13}C NMR of 6-Amino-4-(4-Nitrophenyl)-3-methyl-2,4-dihydropyrano[2,3-c] pyrazole-5-carbonitrile in DMSO.

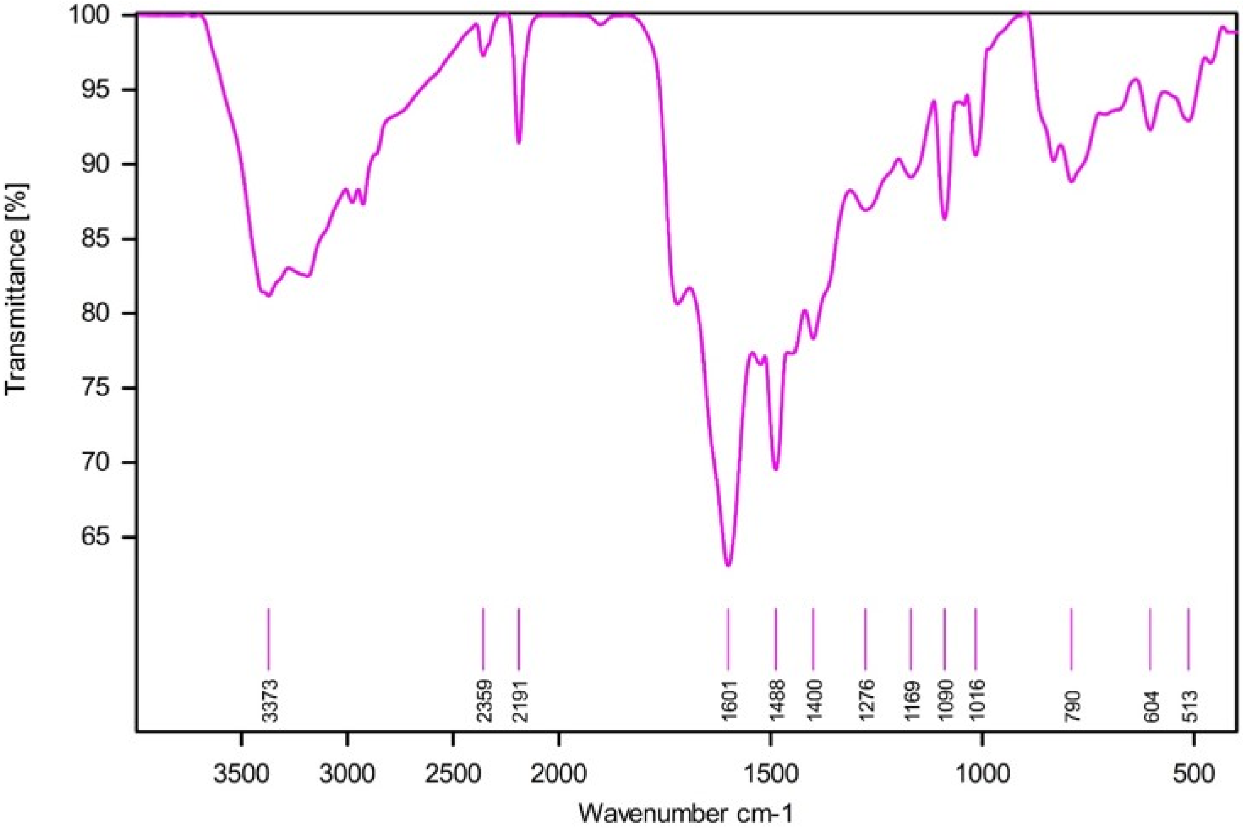

Figure S21. FT-IR of 6-Amino-4-(4-chlorophenyl)-3-methyl-2,4-dihydropyrano[2,3-c] pyrazole-5-carbonitrile in KBr.

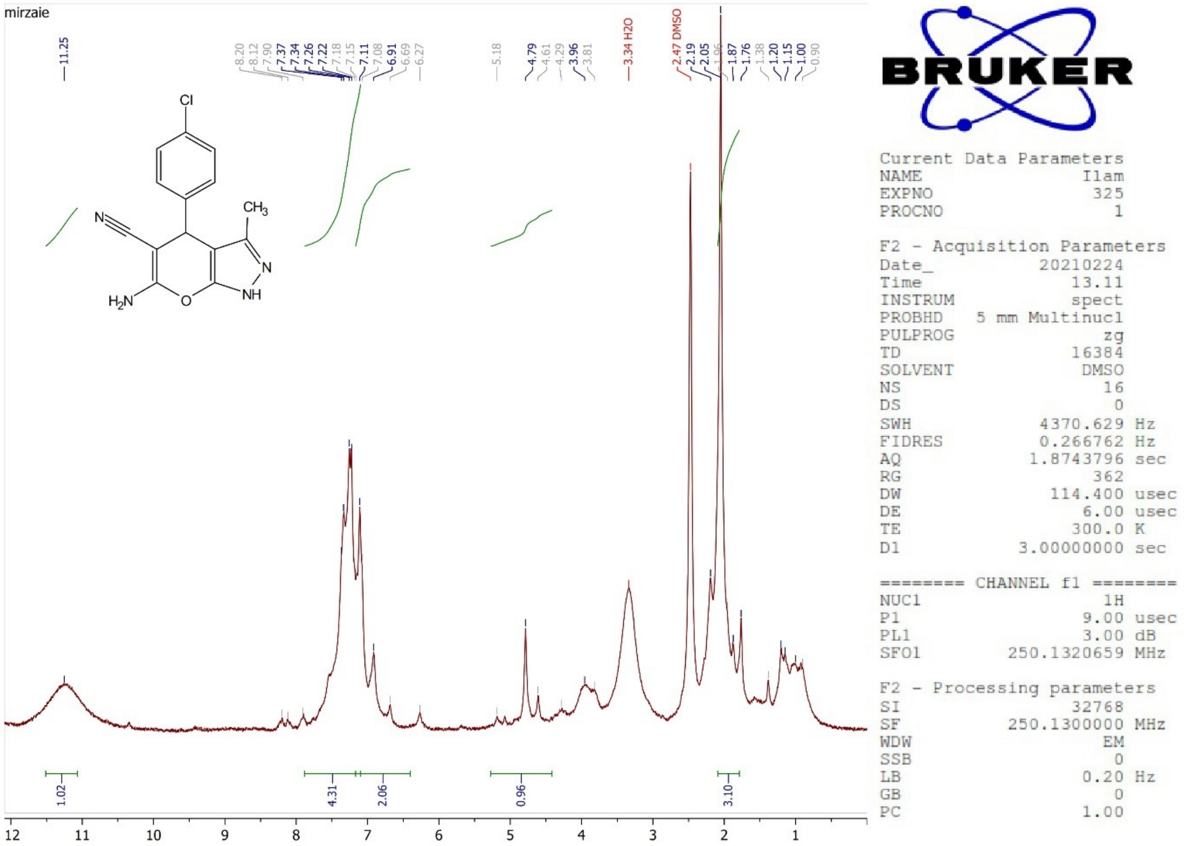

Figure S22. ^{1}H NMR of 6-Amino-4-(4-chlorophenyl)-3-methyl-2,4-dihydropyrano[2,3-c] pyrazole-5-carbonitrile in DMSO.

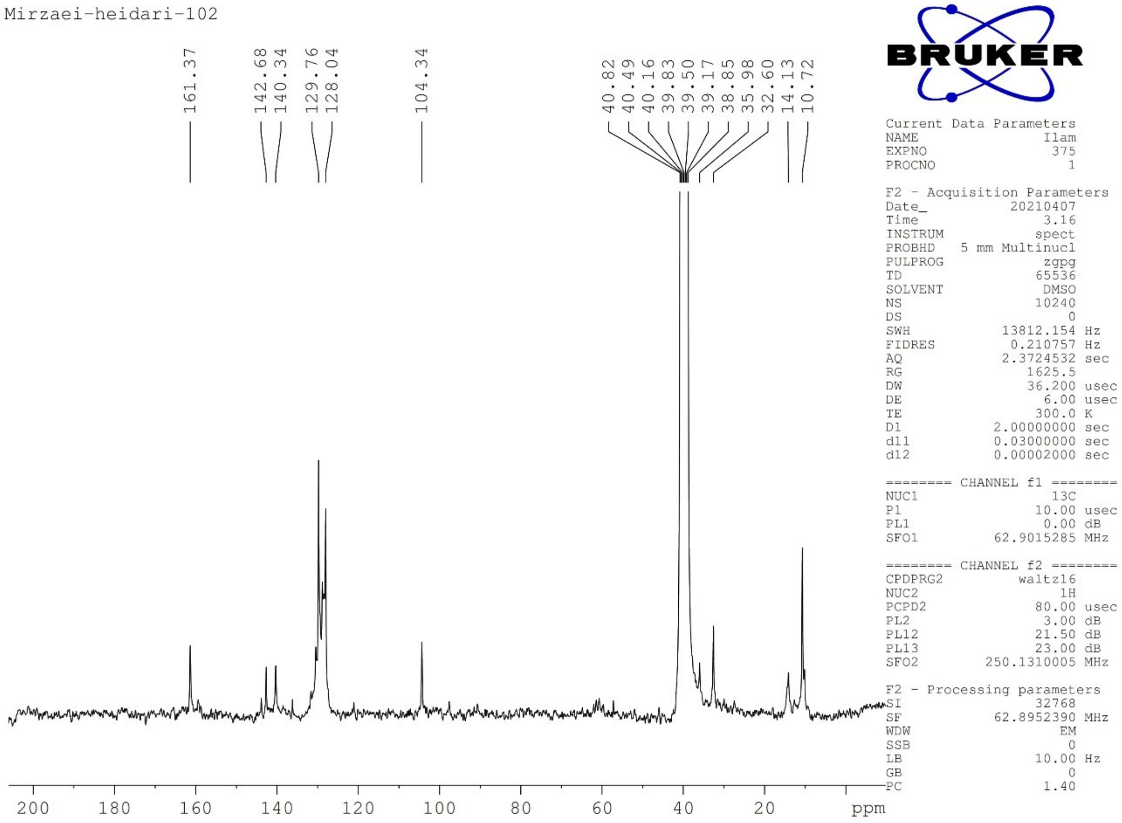

Figure S23. ^{13}C NMR of 6-Amino-4-(4-chlorophenyl)-3-methyl-2,4-dihydropyrano[2,3-c] pyrazole-5-carbonitrile in DMSO.

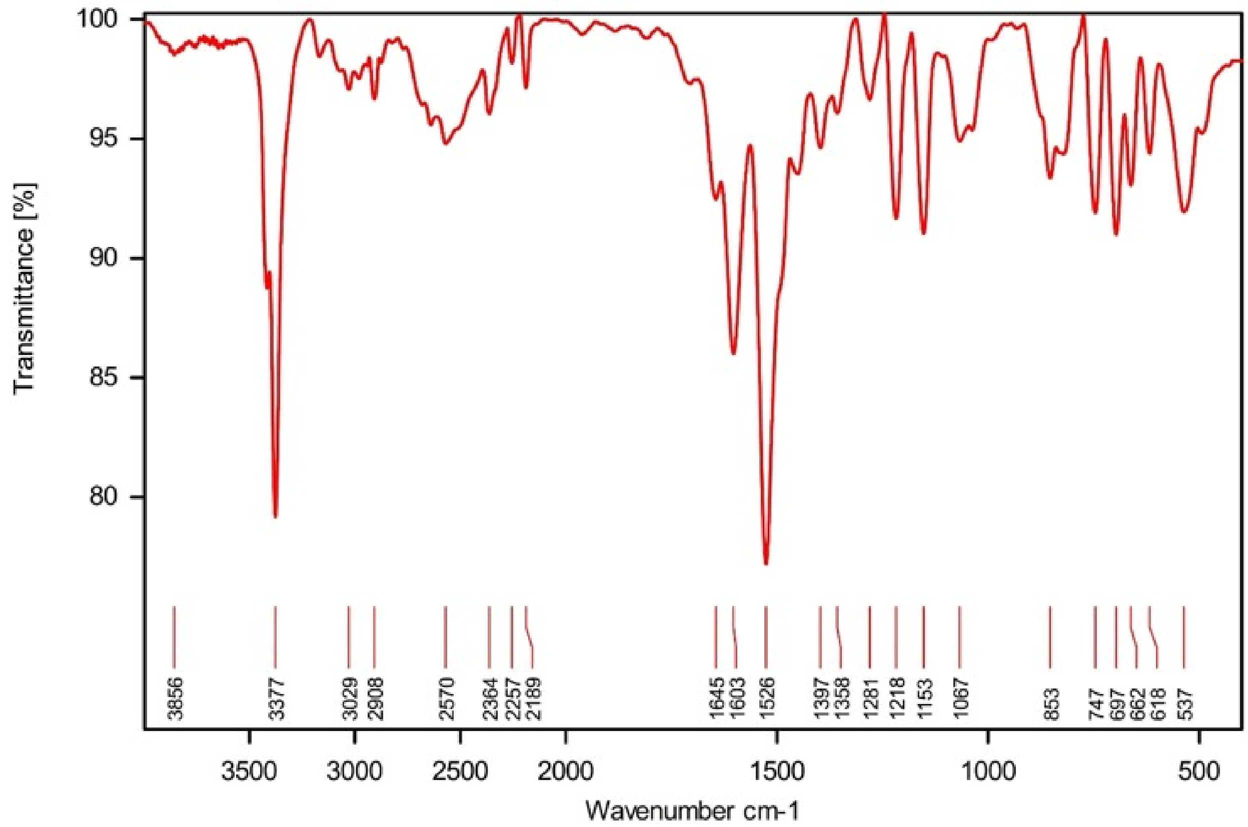

Figure S24. FT-IR of 6-amino-3-methyl-4-phenyl-1,4-dihydropyrano[2,3-c]pyrazole-5-carbonitrile in KBr.

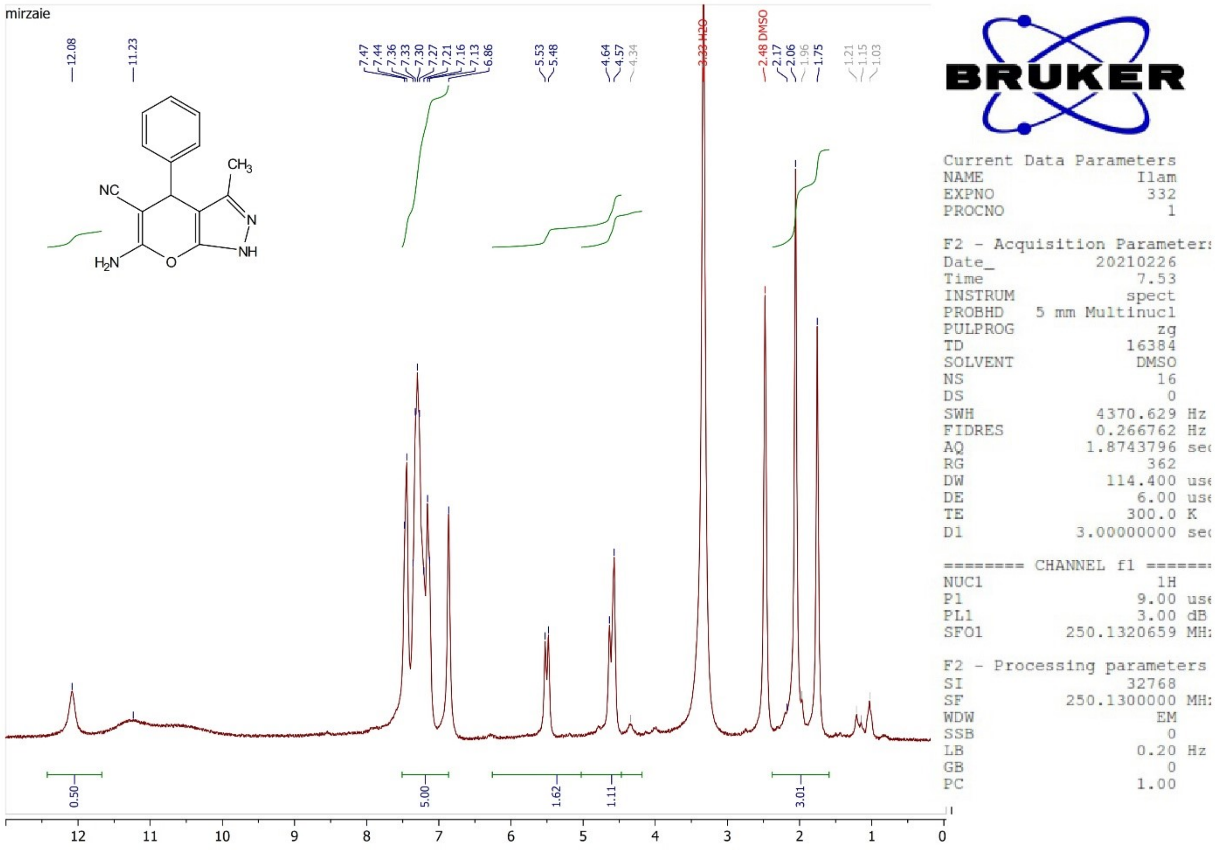

Figure S25. ^{1}H NMR of 6-amino-3-methyl-4-phenyl-1,4-dihydropyrano[2,3-c]pyrazole-5-carbonitrile in DMSO.

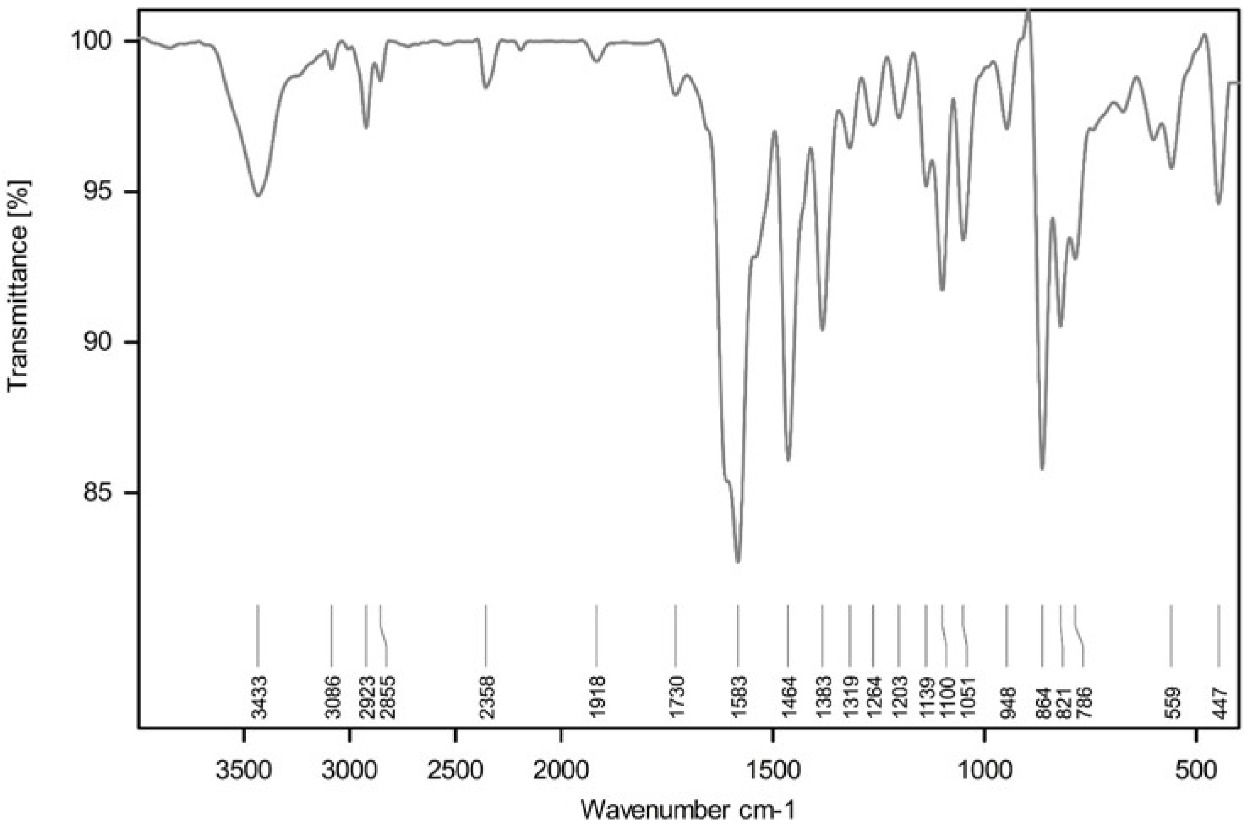

Figure S26. FT-IR of 4-(2,4-chlorophenyl)-3,6,8-trimethyl-4,8-dihydropyrazolo[4',3':5,6]pyrano[2,3-d]pyrimidine-5,7(1H,6H)-dione in KBr.

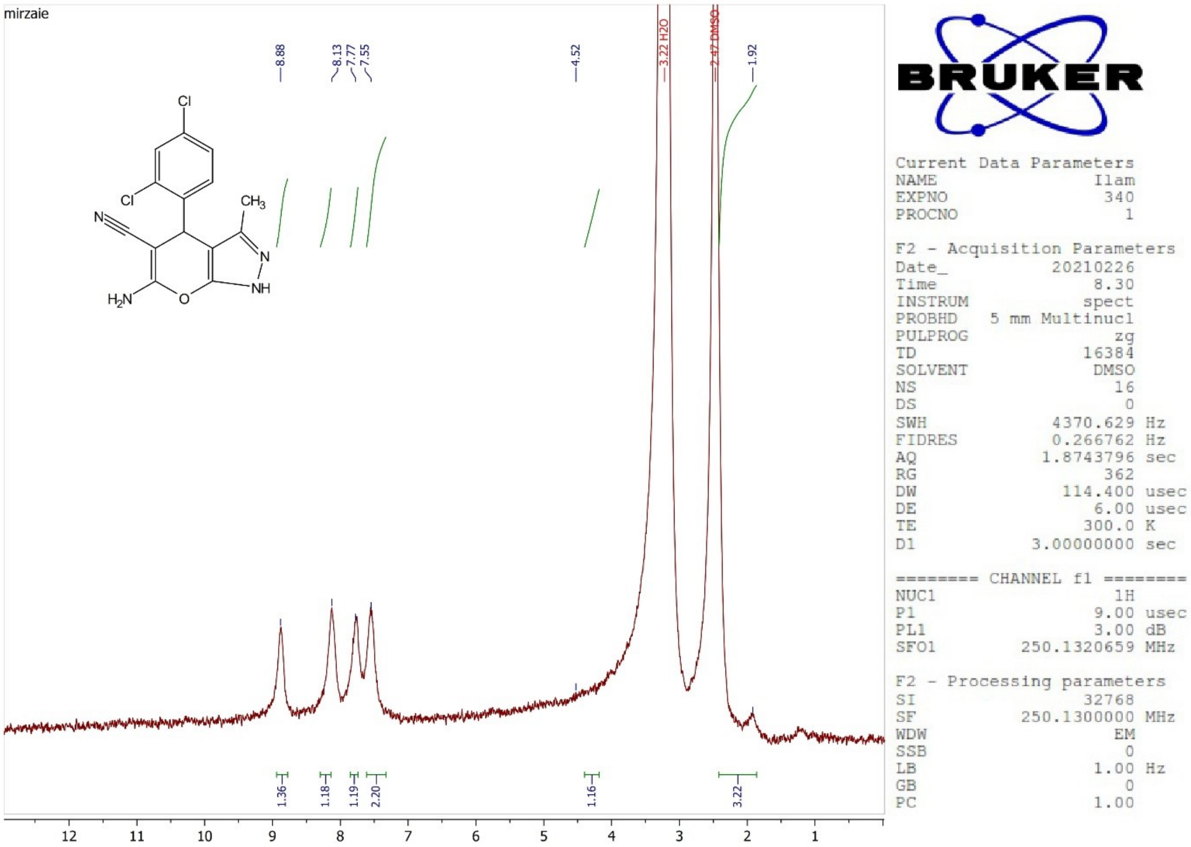

Figure S27. $_1$H NMR of 4-(2,4-chlorophenyl)-3,6,8-trimethyl-4,8-dihydropyrazolo[4',3':5,6]pyrano[2,3-d]pyrimidine-5,7(1H,6H)-dione in DMSO.

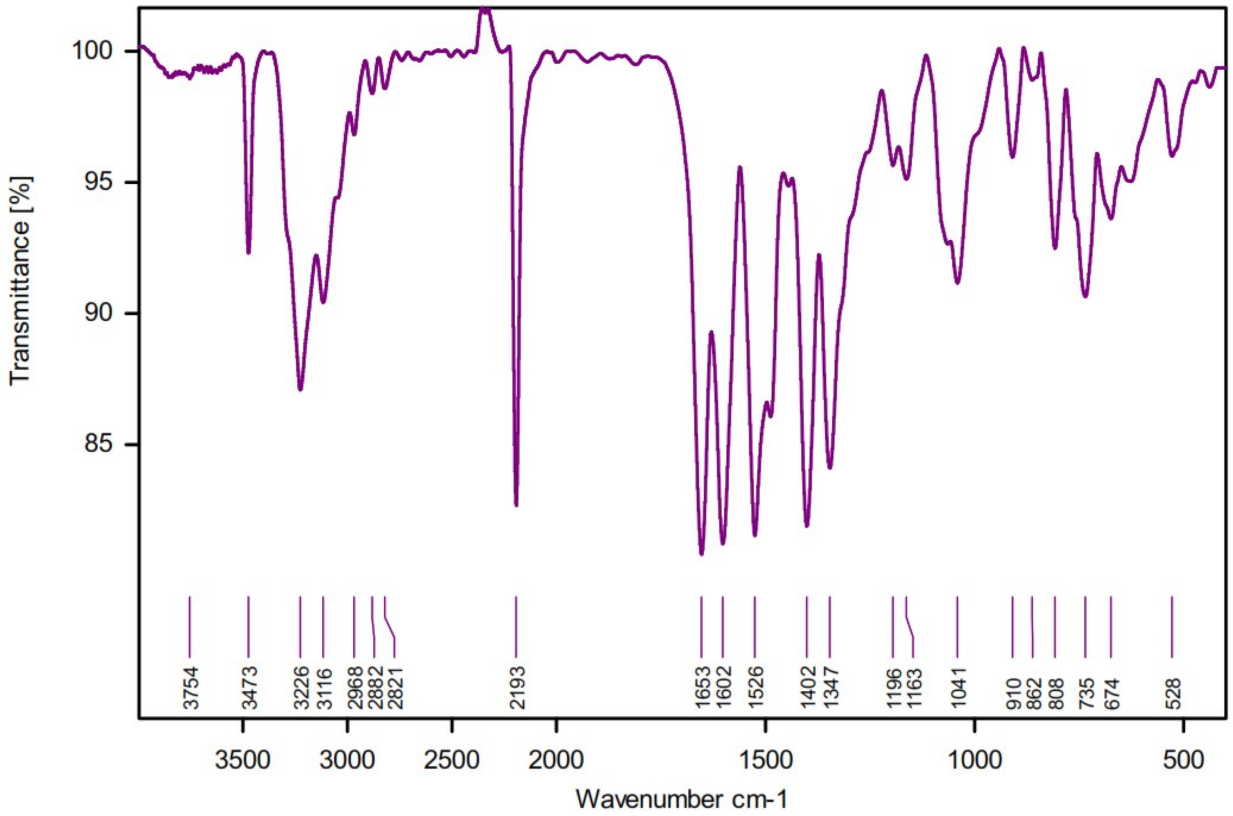

Figure S28. FT-IR of 6-amino-3-methyl-4-(3-nitrophenyl)-1,4-dihydropyrano[2,3-c]pyrazole-5-carbonitrile in KBr.

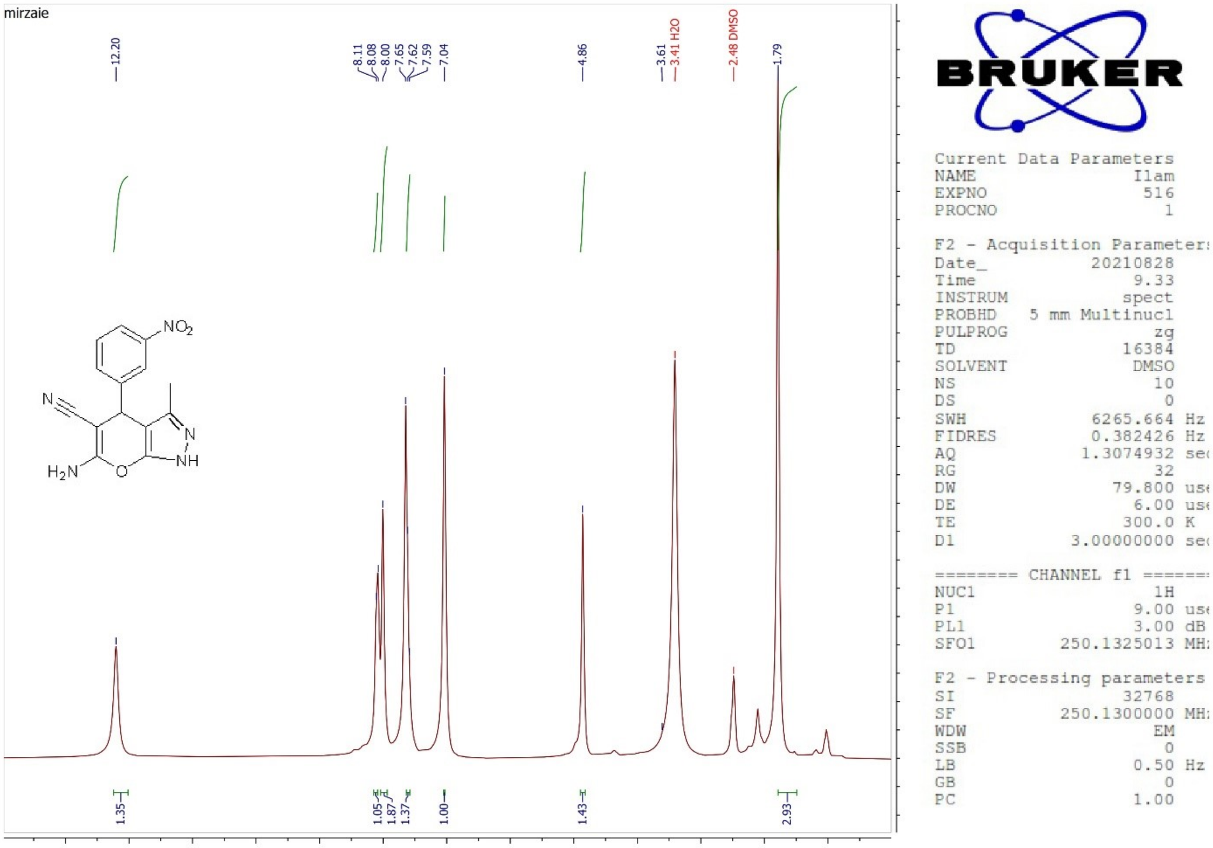

Figure S29. ^{1}H NMR of 6-amino-3-methyl-4-(3-nitrophenyl)-1,4-dihydropyrano[2,3-c]pyrazole-5-carbonitrile in DMSO.

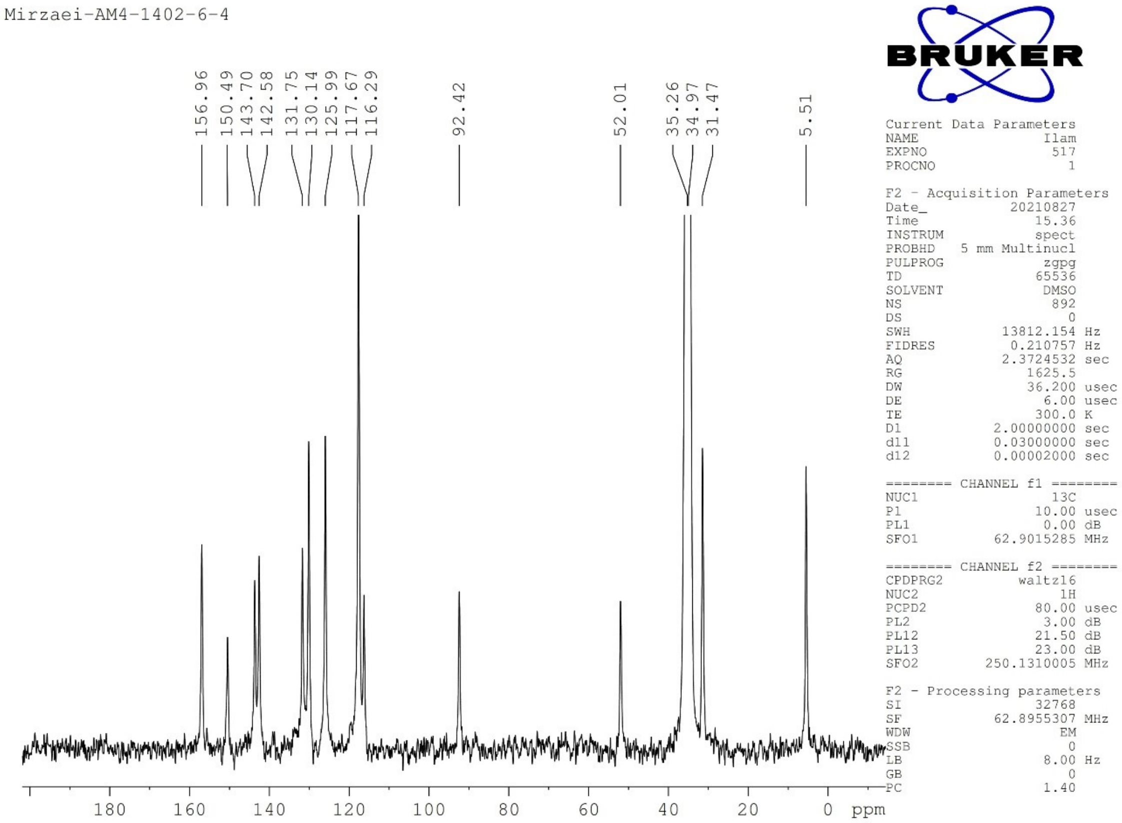

Figure S30. ^{13}C NMR of 6-amino-3-methyl-4-(3-nitrophenyl)-1,4-dihydropyrano[2,3-c]pyrazole-5-carbonitrile in DMSO.

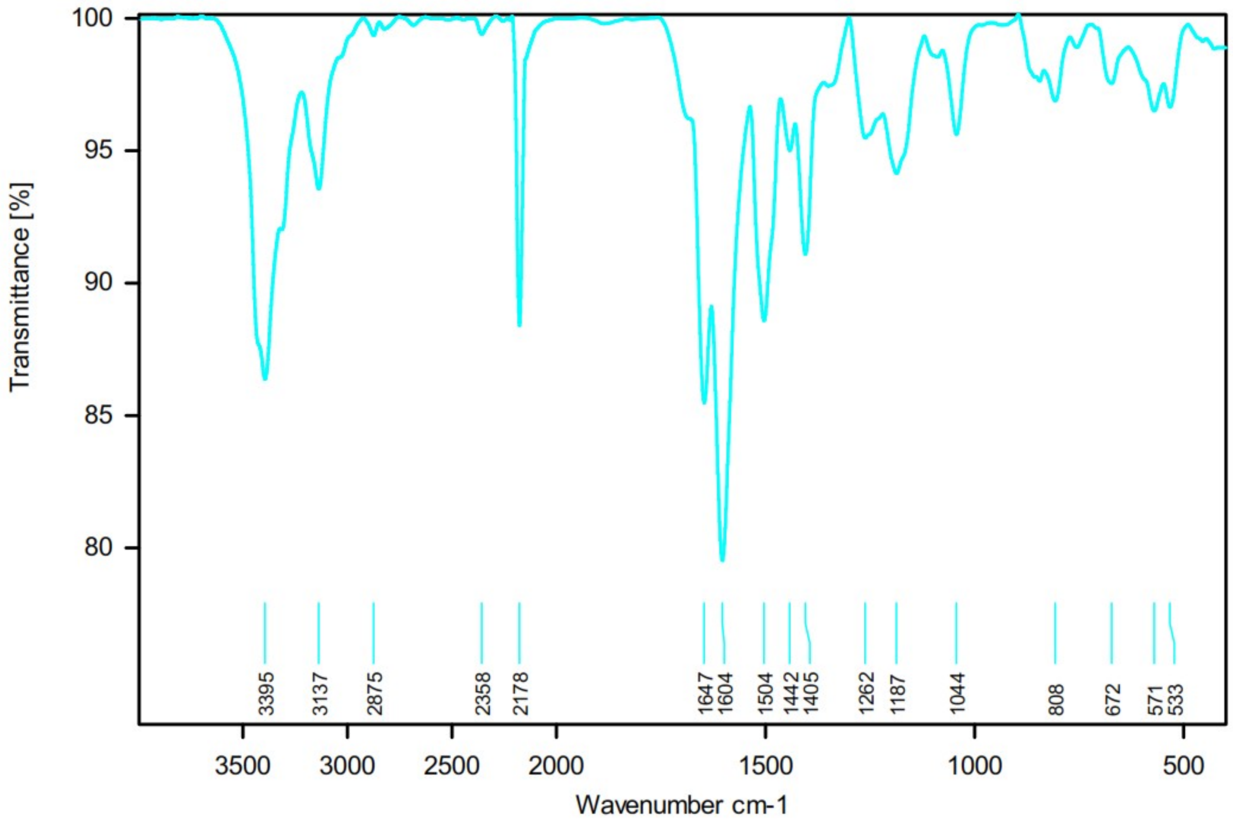

Figure S31. FT-IR of 6-amino-4-(4-hydroxyphenyl)-3-methyl-1,4-dihydropyrano[2,3-c]pyrazole-5-carbonitrile in KBr.

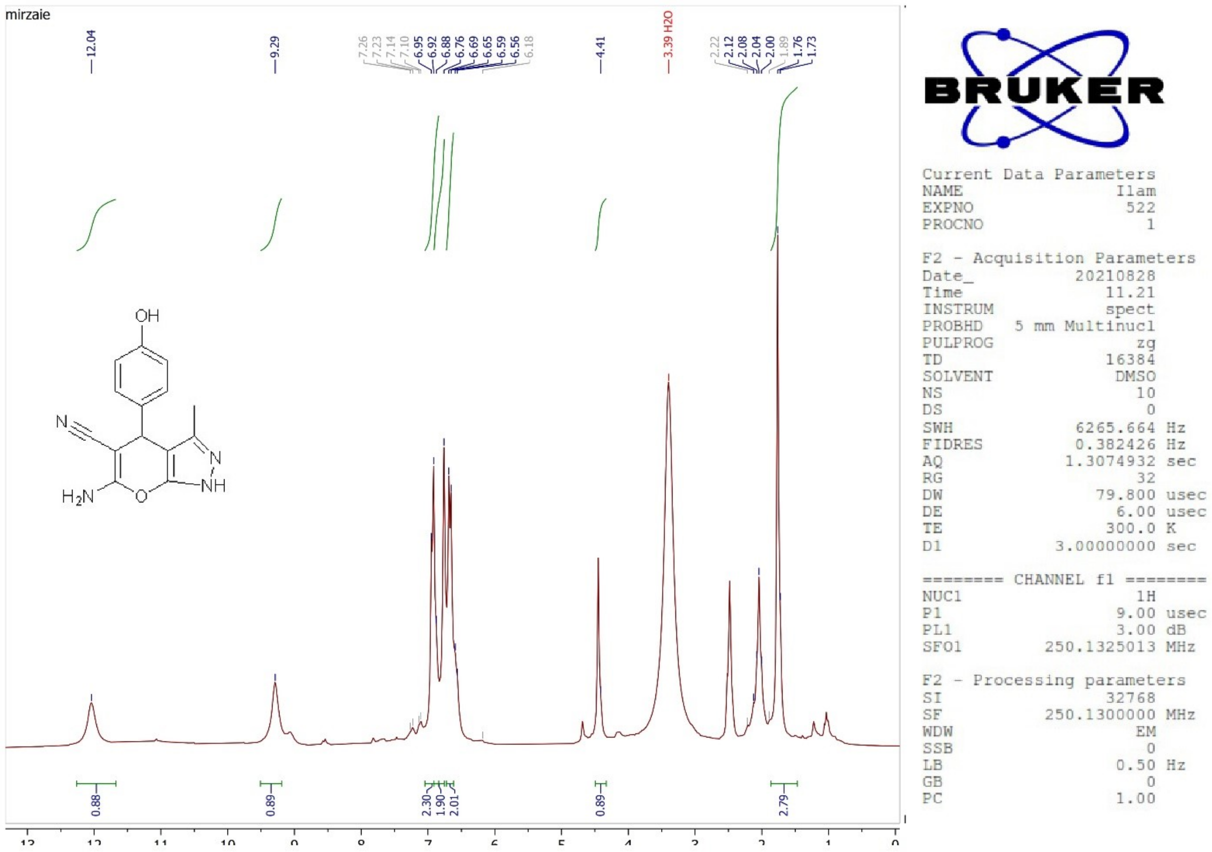

Figure S32. ^{1}H NMR of 6-amino-4-(4-hydroxyphenyl)-3-methyl-1,4-dihydropyrano[2,3-c]pyrazole-5-carbonitrile in DMSO.

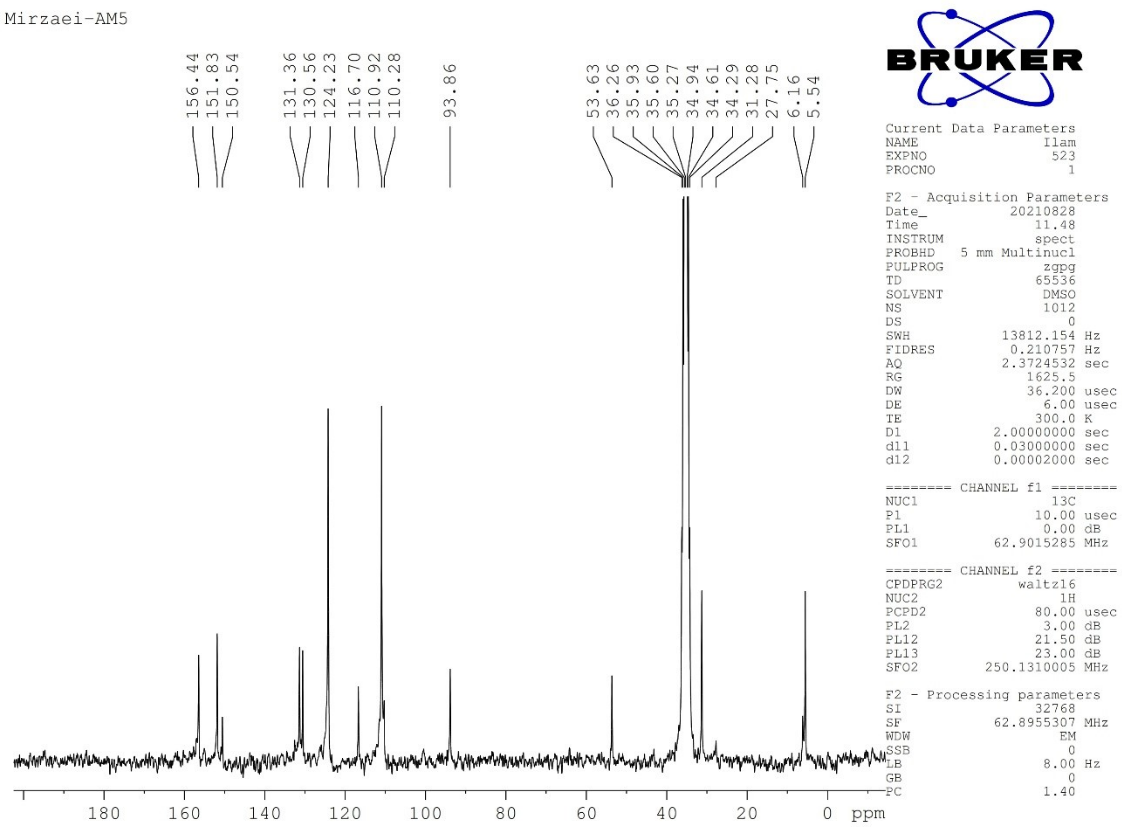

Figure S33. ^{13}C NMR of 6-amino-4-(4-hydroxyphenyl)-3-methyl-1,4-dihydropyrano[2,3-c]pyrazole-5-carbonitrile in DMSO.
